# Albumin-seeking dyes with adjustable assemblies *in situ* enable programmable imaging windows and targeting tumor imaging

**DOI:** 10.7150/thno.92991

**Published:** 2024-04-15

**Authors:** Yijing Du, Jiajun Xu, Tianyang Han, Zijian Jiang, Yuewei Zhang, Jia Li, Xiaoyuan Chen, Shoujun Zhu

**Affiliations:** 1 Joint Laboratory of Opto-Functional Theranostics in Medicine and Chemistry, First Hospital of Jilin University, Jilin University, Changchun 130021, P.R. China.; 2 State Key Laboratory of Supramolecular Structure and Materials, Center for Supramolecular Chemical Biology, College of Chemistry, Jilin University, Changchun 130012, P.R. China.; 3 School of Chemistry and Pharmaceutical Engineering, Jilin Institute of Chemical Technology, Jilin 132022, P.R. China.; 4 Departments of Diagnostic Radiology, Surgery, Chemical and Biomolecular Engineering, Biomedical Engineering, Yong Loo Lin School of Medicine and College of Design and Engineering, National University of Singapore, 117597, Singapore, Singapore.; 5 Clinical Imaging Research Centre, Centre for Translational Medicine, Yong Loo Lin School of Medicine, National University of Singapore, Singapore 117599, Singapore.; 6 Nanomedicine Translational Research Program, NUS Center for Nanomedicine, Yong Loo Lin School of Medicine, National University of Singapore, Singapore 117609, Singapore.

**Keywords:** Dynamic albumin binder, Programmable imaging windows, Dye@albumin nanoparticles, Protein pocket-escaping design, Tumor-targeting dyes

## Abstract

Cyanine dyes are widely used organic probes for *in vivo* imaging due to their tunable fluorescence. They can form complexes with endogenous albumin, resulting in enhanced brightness and photostability. However, this binding is uncontrollable and irreversible, leading to considerable nonspecific background signals and unregulated circulation time.

**Methods:** Here, we connect varying numbers of 4-(4-iodophenyl) butanoic acid (IP) as albumin-binding moieties (ABM) to the cyanine dye, enabling dynamic and controllable binding with albumin. Meanwhile, we provide a blocking method to completely release the dye from covalent capture with albumin, resulting in specific targeting fluorescence. Furthermore, we evaluate the pharmacokinetics and tumor targeting of the developed dyes.

**Results:** The engineered dyes can dynamically and selectively bind with multiple albumins to change the *in situ* size of assemblies and circulation time, providing programmable regulation over the imaging time window. The nucleophilic substitution of meso-Cl with water-soluble amino acids or targeting peptides for IP-engineered dye further addresses the nonspecific signals caused by albumin, allowing for adjustable angiography time and efficient tumor targeting.

**Conclusion:** This study rationalizes the binding modes of dyes and proteins, applicable to a wide range of near-infrared (NIR) dyes for improving their *in vivo* molecular imaging.

## Introduction

Near-infrared (NIR) imaging has become an attractive focus as a convenient tool for multiscale monitoring, ranging from organelles to organisms, and has facilitated clinical translation in areas such as clinical diagnosis and navigation surgery 1-13. Among NIR organic fluorescent dyes, cyanine dyes stand out due to their tunable wavelength, efficient synthesis protocol, and bright fluorescence 14-16. However, most cyanine dyes suffer from poor water solubility and fluorescence quenching through the aggregation-caused quenching effect (ACQ) 17, which significantly affects their biocompatibility and brightness. These fundamental challenges can be addressed through two approaches. Firstly, dyes can be encapsulated in mPEG-DSPE lipids or other water-soluble polymers to form nanoparticles 18-22 or linked polyethylene glycol (PEG) chains 23-25, allowing the dyes to assemble into nanoaggregates in buffer solution or blood. However, this strategy can only partially restore the brightness of the dyes, and the biosafety of PEG has been questioned in recent years 26-28. Humans can develop antibody responses to PEG, resulting in potentially dangerous allergic reactions and potentially limiting the repeated administration of PEG-containing formulations.

The other method involves loading or enveloping the dye with proteins to form a nanoprobe, which relieves the inherent fluorescence quenching 29-33. Common cyanine dyes with meso-Cl, such as IR-780, IR-783, or IR-808, have been reported to rapidly bind with endogenous albumin through a covalent bond, forming stable complexes 31, 34. This binding largely improves brightness and reduces photobleaching due to albumin's hydrophobic pocket restricting twisted intramolecular charge transfer (TICT) and preventing the occurrence of ACQ. Although this method improves biocompatibility and prolongs circulation, the irreversible covalent binding leads to long-term retention and whole-body distribution of dyes *in vivo* due to the widespread presence of albumin in living organisms 35. The nonspecific fluorescence signal limits the application in clinical settings and hampers long-term monitoring of biological events. Moreover, the inherent retention time of dyes *in vivo*, determined by the too-long circulatory half-life of albumin, renders the pharmacokinetics of dyes uncontrollable.

Albumin binding moieties (ABMs) refer to a class of molecular fragments that exhibit a high affinity for albumin and can be used as cargo agents by hitchhiking onto albumin 36, 37. The reversible, non-covalent binding of ABMs to albumin can control pharmacokinetics as intended 38. Examples of ABMs include fatty acids (FAs) 39, 40 and Evans Blue (EB) 41-43. Another important class of ABMs is the 4-(4-iodophenyl) butanoic acid (IP) derivatives proposed by Neri's group, initially discovered from a DNA-encoded chemical library 44. These binders have dissociation constants (Kd) in the low micromolar range, making them suitable as albumin binders 45, 46. Currently, the covalent binding strategy between dyes and albumin lacks universality, and only dyes of a certain size can be embedded into the cavity of albumin. Conversely, ABM-modified dyes have albumin affinity that is not limited by size. We hypothesize that cyanine dyes modified by multiple ABMs can increase the probability of binding with proteins, leading to the formation of dynamic multi-albumin binding *in situ*. This has the potential to regulate the blood half-life of dyes or program the imaging time window 47.

**Scheme 1:**
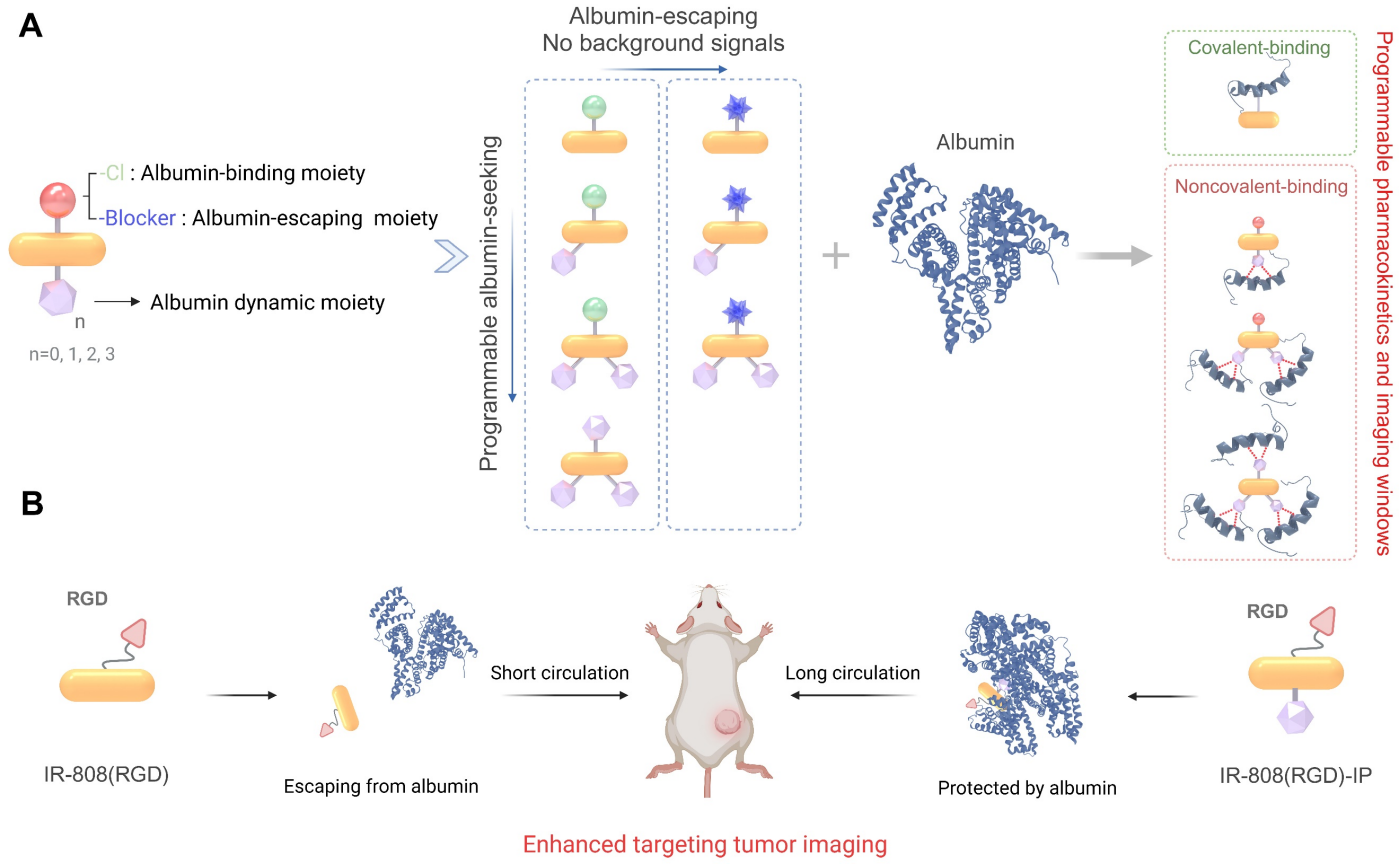
(A) The binding behavior of different dye moieties with albumin, including covalent binding between meso-Cl moiety and site-specific cysteine group in albumin, dynamic interactions between IP moiety and the hydrophobic pocket of albumin, as well as albumin-escaping moiety which could prevent the covalent interaction between dye and albumin. (B) IR-808(RGD)-IPn showed high-performance in tumor targeting.

The non-specific fluorescence of cyanine dyes caused by interference from albumin is a commonly encountered problem 35, particularly in tumor localization where it can significantly affect the accuracy and contrast of targeted probes. IR-808 is a well-established probe for tumor mapping 16, 48-50, but its high skin signal poses challenges in accurately depicting tumor location and shape. Although blocking the albumin binding site (-Cl) can prevent non-specific fluorescence by ensuring complete protein escape, it also leads to rapid dye metabolism, leading to a very short imaging window. In this study, we propose two decoration strategies for IR-808 that rationalize the binding behavior between traditional commercial cyanine dyes and albumin (**Scheme 1**). The first approach involves occupying the meso-Cl position to prevent irreversible covalent binding between proteins and dyes, resulting in extremely low skin signal and avoiding the skin leakage behavior of dyes 51, 52. The second strategy is to connect a variable number of IPs to blocked IR-808, enabling dynamic binding with multi-albumin through non-covalent interactions and providing controllable pharmacokinetics. This dynamic non-covalent binding thereby overcomes the ultrafast blood metabolism of albumin-escaping dyes. These strategies combine to effectively regulate dye pharmacokinetics *in vivo* and allow for precise tuning of the imaging time window, as demonstrated in lymph node imaging in mice by constructing multi-level lymph node time windows of varying durations. Furthermore, replacing blockers with tumor-targeting peptides on the dyes can significantly enhance their tumor-seeking ability, as the IP moieties have a long blood half-life. Our methodology offers an effective approach to better utilize fluorescent probes for *in vivo* imaging and maximize the targeting imaging effect.

## Results and Discussion

### Optimization of Molecular Fluorophores Beneficial for Adjustable Assemblies with Albumin

**Figure 1:**
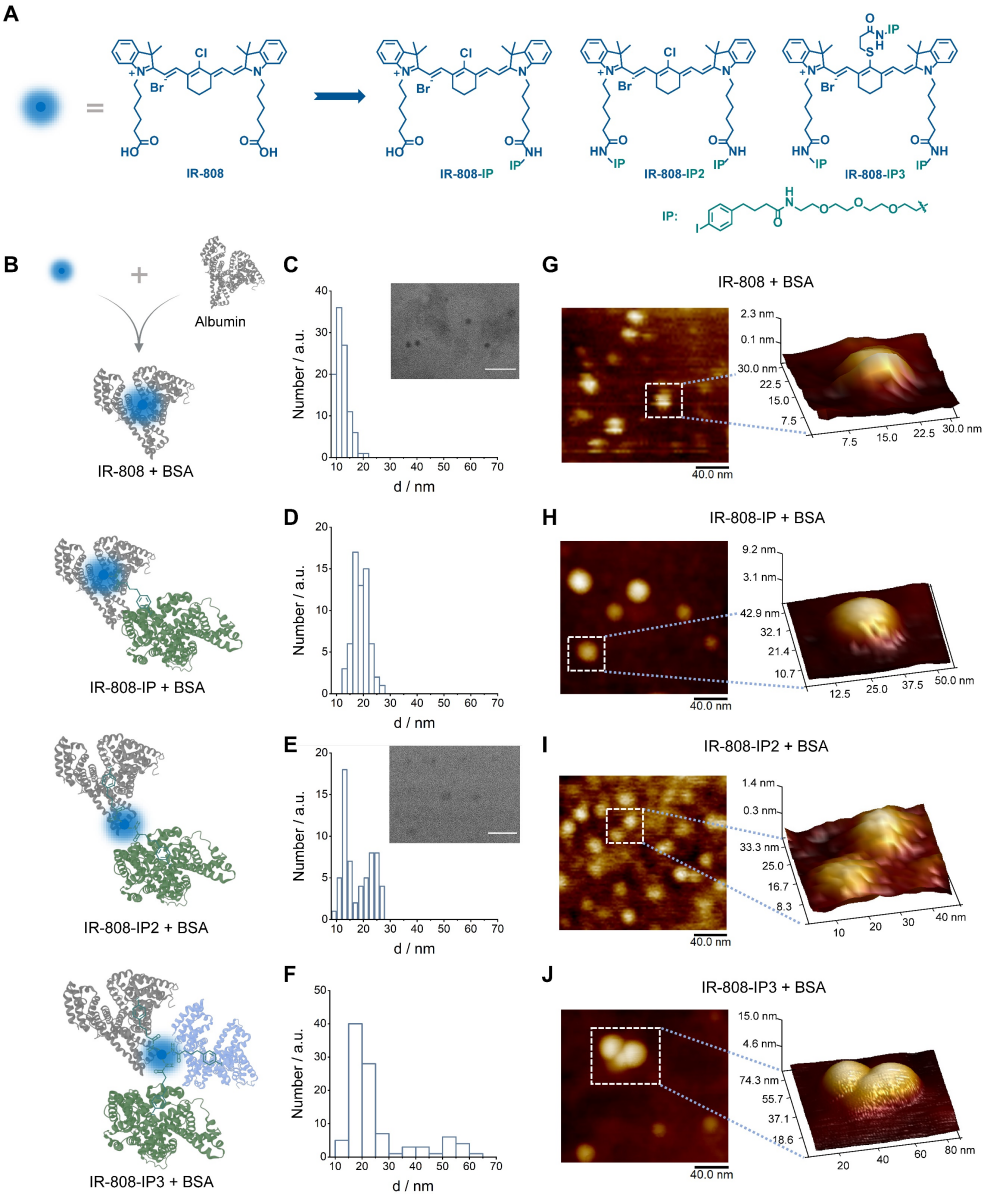
**Chemical structures of cyanine dyes and their interaction with albumin.** (A) Chemical structures of four cyanine dyes: IR-808, IR-808-IP, IR-808-IP2, and IR-808-IP3. (B) Schematic diagram of possible binding modes between different dyes and albumin. (C-F) Transmission electron microscopy (TEM) images of IR-808 (C), IR-808-IP (D), IR-808-IP2 (E), and IR-808-IP3 (F) in BSA solution at a concentration of 200 μM with a dye-to-BSA ratio of 1:1. Scale bar : 50 nm. (G-J) Atomic force microscopy (AFM) images of IR-808 (G), IR-808-IP (H), IR-808-IP2 (I), and IR-808-IP2 (J) in BSA solution at a concentration of 1 μM with a dye-to-BSA ratio of 1:1.

We first designed and synthesized a series of molecules (IR-808, IR-808-IP, IR-808-IP2, and IR-808-IP3) by coupling various quantities of 4-(4-iodophenyl) butanoic acid derivatives (IP) with IR-808 and differentiated them based on the number of connected IPs (**Figure 1A**). The incorporation of a mini-PEG chain effectively reduced the aggregation behavior of long alkane chains by increasing the water solubility of the molecule. This type of modification did not alter the excellent NIR fluorescence performance of IR-808, and the wavelengths of absorption and fluorescence emission remained unchanged. However, as the number of modified IPs increased, both spectral curves tended to flatten out in the bovine serum albumin (BSA) solution (**Figure S1**), suggesting that the interaction between albumin and the molecules has been altered.

To investigate the interaction between IP-modified IR-808 and albumins, we employed transmission electron microscopy (TEM) to examine the bound compounds (Figure 1C-F). Both IR-808, IR-808-IP, IR-808-IP2, and IR-808-IP3 were incubated with BSA at a molar ratio of 1:1. TEM images and distribution analysis revealed that the IR-808 and IR-808-IP in BSA formed uniformly sized particles that followed a Gaussian distribution, similar to single BSA (Figure 1B), confirming the formation of a stable monomer complex between IR-808 and BSA. It is worth noting that there was a slight increase in particle size in the size statistics of the mixture of IR-808-IP and BSA, which was speculated to be caused by the non-covalent interaction between IP groups in IR-808-IP and BSA (Figure 1B). In Figure 1D and 1E, a portion of larger-sized particles were observed. Notably, IR-808-IP2 and IR-808-IP3, as dyes with multiple albumin-binding moieties, were able to induce the dimerization of albumin. We further determined the morphological structures of the complex present in the system by atomic force microscopy (AFM) (Figure 1G-J). As expected, the majority of IR-808@BSA complex and IR-808-IP@BSA complex appeared as individual spheres, while the mixture of IR-808-IP2 and albumin showed the existence of hourglass-shaped particles. Particularly, some obvious trimers were observed in the mixture of IR-808-IP3 and albumin. The size obtained by TEM and AFM were consistent within acceptable error ranges, supporting our hypothesis that the addition of IP can provide albumin binding sites for the dye to form single, di-albumin, or tri-albumin complexes (Figure 1B). At the same time, the introduction of IP modification also increases the dynamic size of a single molecule in the solution, making it difficult for meso-Cl to covalently bind to proteins (Figure S2 and Table S1).

### The Programmable Imaging Windows of Lymph Nodes

**Figure 2:**
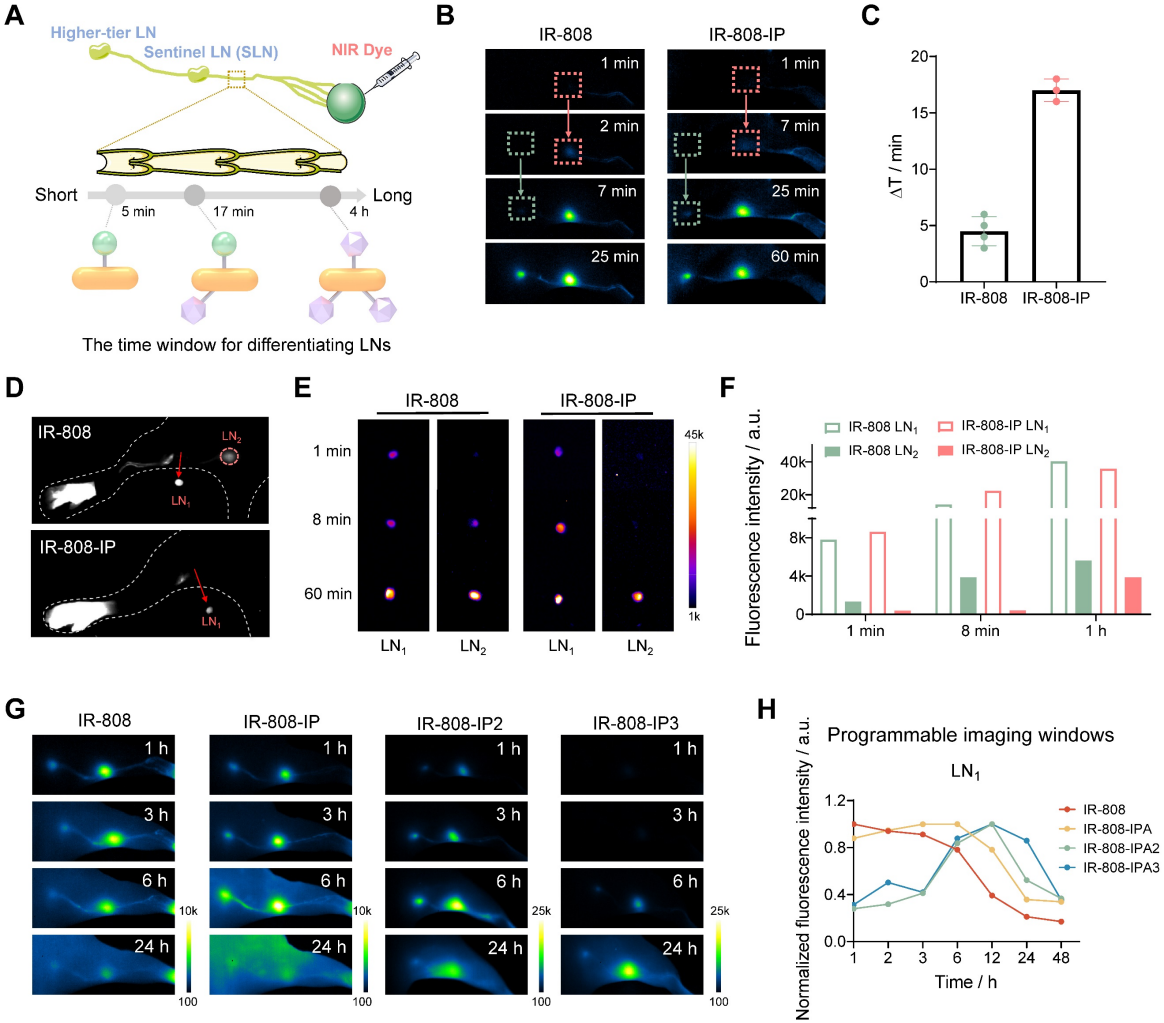
**The imaging time window of IR-808-IP(n) for distinguishing LNs.** (A) Schematic illustration of the time window for differentiating LNs. (B) Significant difference in NIR-II imaging time points for lighting LN_1_ and LN_2_ after the injection of IR-808 (left) and IR-808-IP (right) into the mice's right footpad without pressing. Exposure time for IR-808 is 50 ms while for IR-808-IP is 200 ms. (C) The time for distinguishing LN_1_ and LN_2_ by NIR-II fluorescence brightness of IR-808 (n=4) and IR-808-IP (n=3). (D) NIR-II-guided surgical excision of LN_1_ approximately 5 min post-injection of IR-808 and IR-808-IP. NIR-II imaging (E) and brightness statistics (F) of LN_1_ and LN_2_ *in vitro* after intradermally injecting for 1 min, 8 min, and 60 min without pressure on the mice's footpad. (G) NIR-II fluorescence imaging of mice lymph nodes at key time points after the injection of dyes. Exposure time for IR-808 is 20 ms, for IR-808-IP and IR-808-IP2 are 60 ms, for IR-808-IP3 is 100 ms. (H) Normalized NIR-II fluorescence brightness statistics of LN_1_ over time from (G). Laser excitation: 808 nm with 65 mW/cm^2^; Filter: 1000 /1100 nm long-pass filters; All footpad injection doses were 25 μL (200 μM). LN_1_: popliteal LN; LN_2_: sacral LN.

We evaluated the ability of different dyes to label lymph nodes (LNs) by intradermally injecting them into the foot pads of mice. It has been reported that the intra-lymphatic pharmacokinetics of nanoparticles is related to particle size 53, 54. We expected dyes with multiple IPs to dynamically capture more albumin and form larger complexes, which would alter the speed of dye movement in lymphatic vessels and selectively locate the sentinel lymph node (SLN), rather than rapidly spreading to higher-tier lymph nodes and other tissues, thus avoiding unintended resection of normal LNs (**Figure 2A**). Surprisingly, the illumination of LNs occurred in an organized manner, and the time window for differentiating LNs injected with IR-808-IP was longer compared to IR-808. The popliteal LN, serving as the SLN, was first illuminated after 7 min of injecting IR-808-IP, while the sacral LN was illuminated as a distant LN after 25 min (**Figure 2B**). This differed from the imaging window of 2 min (popliteal LN) and 7 min (sacral LN) for IR-808. After calculating the imaging time differences in multiple groups (n ≥3) of experimental data to exclude individual differences, a significant difference was observed in the selected groups (**Figure 2C**). The time interval generated by IR-808-IP can reach approximately 17 min, which prevented rapid dye leakage to the higher-tier LNs, thus influencing the location of SLNs in more complex organisms and providing sufficient time for accurate SLNs removal. LN excision was performed 5 min after injection, and the NIR fluorescence brightness of the sacral LN was higher when injected with IR-808 compared to IR-808-IP (**Figure 2D**). *In vitro* brightness comparison of leg lymph node removal at different time points after injection was conducted to further validate that the sacral LN had almost no fluorescence accumulation in the short term (**Figure 2E-F**). In the aforementioned experiments, the mice were under continuous anesthesia, and no external pressure was applied to their footpads after injection. Due to the slower lymphatic metabolism, no NIR fluorescence from multiple lymph nodes was observed within a 2 h observation period for IR-808-IP2 and IR-808-IP3. Weak fluorescence signals were only observed in the popliteal LN of IR-808-IP3 injected mice after 80 min (**Figure S3**).

To further compare the differences between dyes, we conducted a longer observation experiment. Anesthetized mice were allowed to move freely after injection, with anesthesia only performed at the observation time points. The results are presented in Figure 2G. IR-808 was rapidly metabolized in the lymphatic system, making it almost impossible to distinguish LNs and lymphatic vessels 24 h later, which is not conducive to long-term observation of the lymphatic system. In contrast, IP-modified molecules exhibited significantly longer lymph node labeling time. The brightness statistics of the popliteal LNs revealed that the brightness of lymph nodes reached its peak value at different time points (Figure 2H). Notably, IR-808-IP2 and IR-808-IP3 had later lighting time windows, with the brightness reaching its maximum value at 12 h. In addition, the sciatic LN lit up by IR-808-IP2 after only 1 hours post-injection (Figure S4), whereas the sciatic LN was found to light up by IR-808-IP3 after 6 h post-injection, providing an extended time window for lymph node visualization.

### The Pharmacokinetics and the Properties of the Dyes

**Figure 3:**
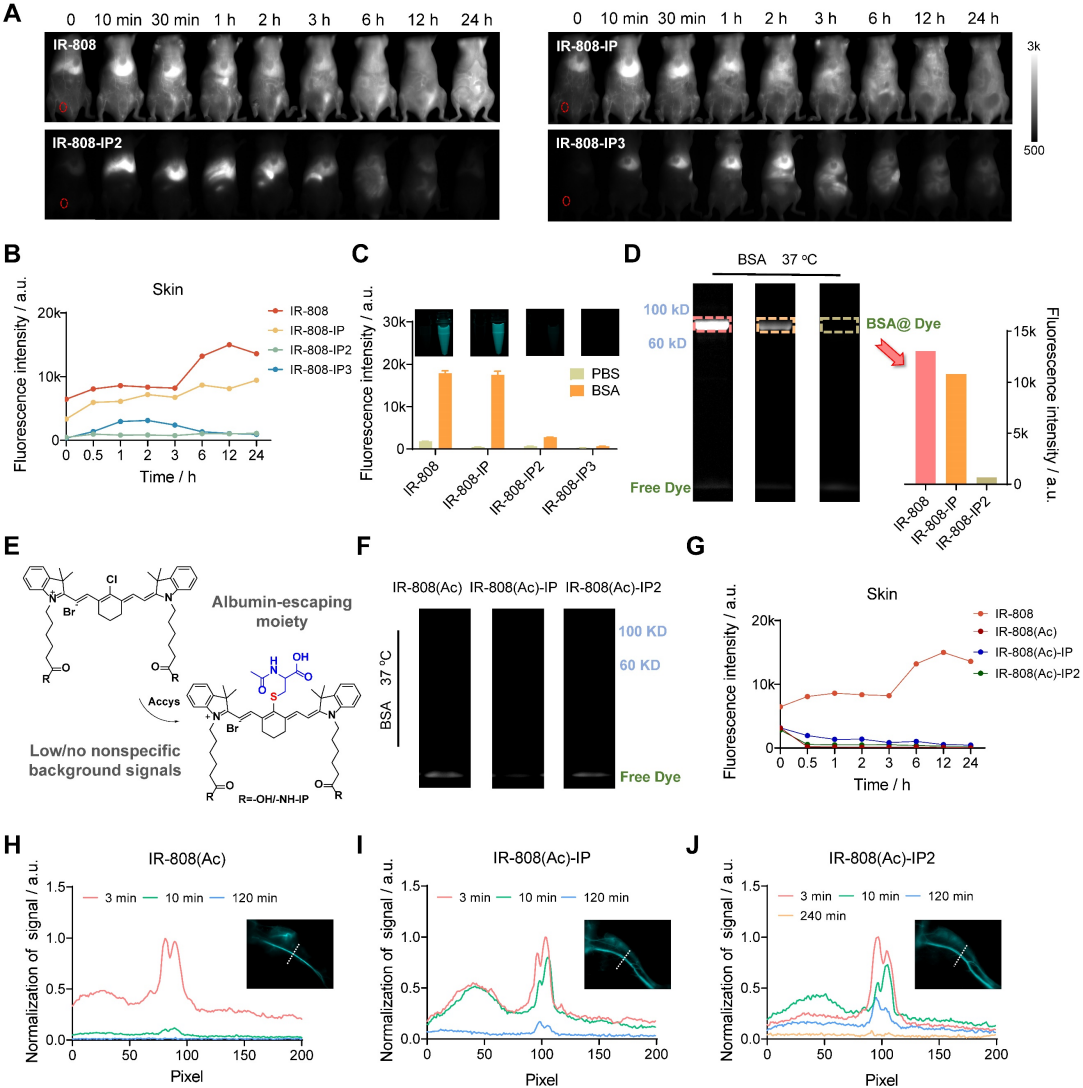
** The pharmacokinetics of IR-808-IP(n) dyes as well as the synthesis and properties of the albumin-escaping IR-808(Ac)-IP(n).** (A) Whole-body NIR-II imaging of mice 0-24 h after tail vein injection of IR-808, IR-808-IP, IR-808-IP2, and IR-808-IP3. The red dotted circles are regions of interest (ROIs) of skin. Over 1100 nm collection; Injection dose: 200 μL (200 μM); Exposure time for IR-808-IP3 is 100 ms, others are 30 ms. (B) NIR-II fluorescence signal statistics of mouse skin within 24 h after tail vein injection. (C) Solution brightness of dyes in PBS and BSA and corresponding near-infrared tube images. Incubation at 37 °C for 2 h with an equal molar mixture; Concentration: 10 μM; Over 1100 nm collection; Exposure time: 3 ms. (D) Electrophoresis analysis of dyes in BSA solution and brightness statistics of albumin bands. Over 1100 nm collection; Exposure time: 50 ms. (E) Synthesis procedure of albumin-escaping dyes: IR-808(Ac), IR-808(Ac)-IP, and IR-808(Ac)-IP2. (F) Electrophoresis analysis of dyes in BSA solution (no albumin bands at their corresponding molecular weights were observed). (G) NIR-II fluorescence brightness statistics of mouse skin within 24 h after tail vein injection of IR-808(Ac), IR-808(Ac)-IP, and IR-808(Ac)-IP2. Over 1100 nm collection; Injection dose: 200 μL (200 μM). (H-J) Normalized cross-sectional fluorescence intensity profiles along the white dotted lines in blood vessels administered by (H) IR-808(Ac), (I) IR-808(Ac)-IP, and (J) IR-808(Ac)-IP2, respectively. Insets are NIR-II imaging of the posterior limb. 808 nm excitation with 100 mW/cm^2^; Over 1300 nm collection; Injection dose: 600 μL (200 μM); Exposure time for IR-808(Ac) is 15 ms, for IR-808(Ac)-IP is 30 ms and for IR-808(Ac)-IP2 is 100 ms.

In order to further investigate the effect of AMB introduction on dyes retention *in vivo*, we conducted a systematic comparison of the metabolic behavior of these dyes and found that all of them underwent hepatobiliary metabolism (**Figure 3A**), with a liver brightness half-life of less than 6 h (**Figure S5**). We confirmed that IR-808 and IR-808-IP exhibited high fluorescence brightness in the skin, while IR-808-IP2 and IR-808-IP3 showed negligible signals (**Figure 3B**). Although IP moiety can dynamically bind to albumin(s), it was suggested that IR-808 and IR-808-IP can rapidly covalently bind with albumin to form stable complexes, while IR-808-IP2 and IR-808-IP3 can avoid being covalently captured by albumin (steric hindrance effect), thus eliminating nonspecific signals. To further validate the binding ability of several dyes to albumin, we incubated dyes and BSA at equal molar concentrations at 37 °C for 2 h. The results showed that the brightness of IR-808 and IR-808-IP increased 10-fold and 36-fold, respectively, in the BSA solution compared to that in the PBS buffer (**Figure 3C**). Previous research has shownthat dyes were captured by albumin hydrophobic cavities, and covalent binding via nucleophilic substitution provided a stable state that confined TICT and contributed to fluorescent enhancement 31. We further examined the efficient covalent binding of albumin with IR-808 or IR-808-IP, which resulted in bright bands at corresponding albumin molecular weights in gel electrophoresis after reacting at room temperature for 2 h (**Figure 3D**). However, the brightness of IR-808-IP2 was slightly restored in BSA (6-fold enhancement compared to PBS) under the same conditions. This can be attributed to the spatial hindrance caused by the extension of the side chain in combination of dynamically bound albumins, which makes it difficult for IR-808-IP2 to enter protein hydrophobic pockets and complete the nucleophilic substitution. In this case, compared with the exposed IP groups, meso-Cl was no longer the optimal binding site. With an incubation temperature increment over 50 °C, IR-808-IP2 could also covalently bind with albumin efficiently (**Figure S6, S7**). The covalent bond formation between IR-808-IP3 and albumin was impossible due to the absence of meso-Cl, resulting in no fluorescence enhancement in both solution and electrophoresis.

As previously confirmed, covalent binding leads to significant fluorescence enhancement, but the nonspecific fluorescence generated by the widespread distribution of albumin can mask the specific signal for certain applications, such as vascular imaging and tumor-targeting imaging 35. Additionally, high skin fluorescence can result in false signals during continuous observation. To address these issues, we further developed absolute albumin-escaping dyes by blocking meso-Cl using a hydrophilic amino acid derivative, acetylcysteine (Ac), as a blocker (**Figure 3E and S8**). After transformation by the blocker, this series of dyes were unable to covalently bind with albumin due to the loss of the active meso-Cl group (**Figure 3F**). We further investigated the basic metabolic behavior of Ac-blocked dyes and found that the fluorescence brightness of the skin was almost unrecognizable (**Figrue 3G and S9A**), and the liver clearance speed varied depending on the number of connected IPs (**Figure S9B**). Although the fluorescence brightness of the dyes did not show significant enhancement after incubation with an equimolar concentration of BSA, the brightness of the Ac-blocked dyes was surprisingly enhanced in the blood vessels after tail vein injection in mice (**Figure 3H-J and S10**). High-resolution vascular imaging was achieved under the NIR-II imaging window using the inherent tail emission of IR-808 dyes, and the vessel-to-skin ratio after injection was not less than 4. As the number of IPs increased from 0 to 2, the vascular imaging time windows could be regulated within the range of 10 min to 4 h, and the fluorescence was not gradually captured by the skin, allowing for better spatiotemporal resolution. The half-life of blood fluorescence after injection of different dyes exhibited significant differences (**Figure S11**), which is consistent with the connected IP numbers. These results verified that the established strategies of transferring covalent binding moiety to dynamic binding groups between dyes and albumin are effective in regulating the pharmacokinetics of dyes.

### *In Vivo* Tumor-targeting Imaging

**Figure 4:**
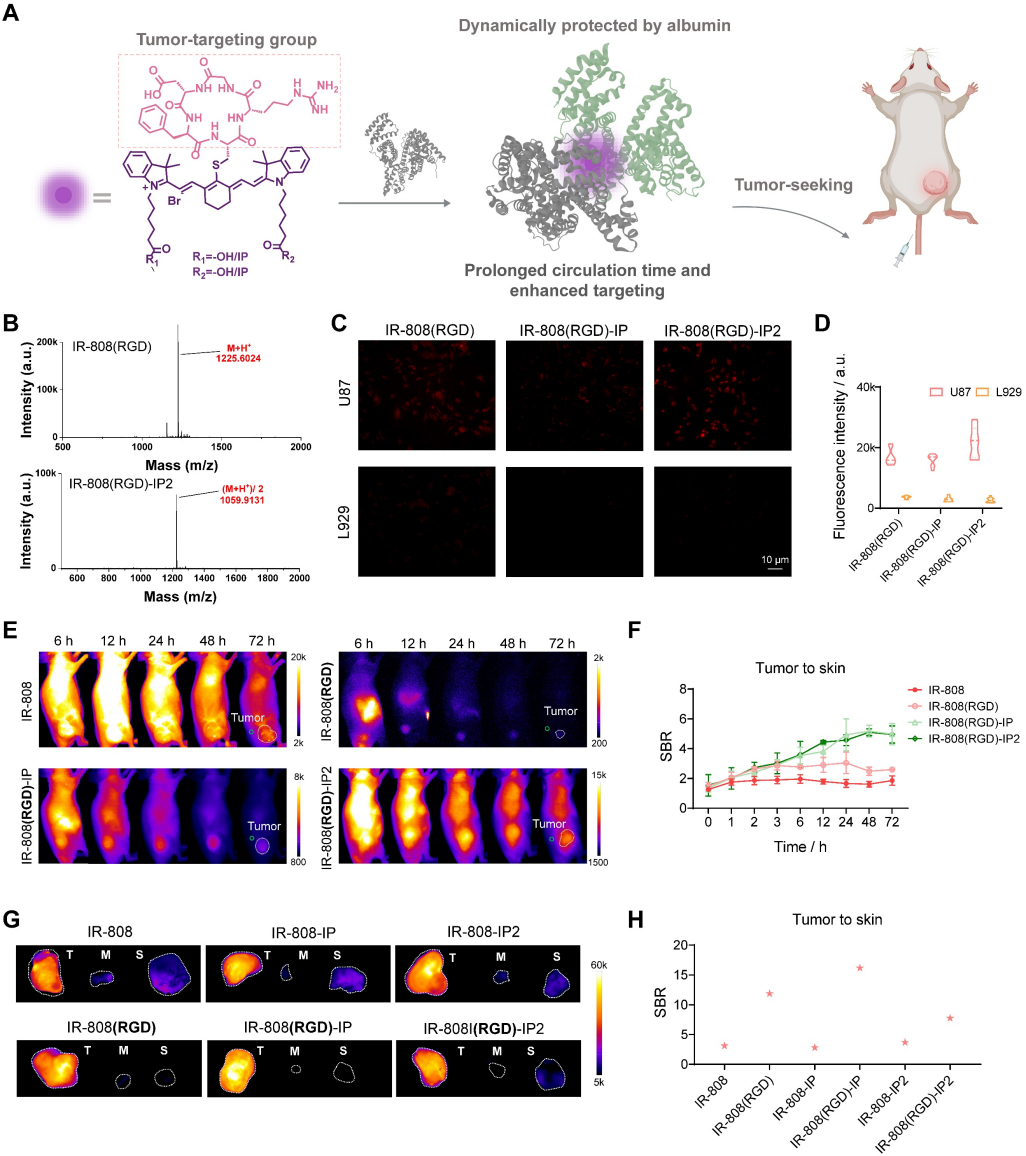
** IR-808(RGD)-IPn enabled high-performance targeting tumor imaging.** (A) Schematic diagram of the targeting molecule structures. (B) HPLC-MS spectra of IR-808(RGD) and IR-808(RGD)-IP2 demonstrated that RGD was successfully attached to the dye molecules. (C) NIR-II microscopy images of αvβ3-positive U87MG and αvβ3-negative L929 cells incubated in DMEM with dyes. (D) NIR-II fluorescence of U87MG cells and L929 cells (n=10) incubated in DMEM with dyes. 808 nm excitation with 65 mW/cm^2^; Over 1000 nm collection. (E) NIR-II imaging of U87MG tumor-bearing mice injected with IR-808, IR-808(RGD), IR-808(RGD)-IP, and IR-808(RGD)-IP2 over time. The green circles are ROIs of skin surrounding the tumor site, and the white dotted circles are ROIs of tumor. 808 nm excitation with 65 mW/cm^2^; Over 1100 nm collection; Injection dose: 200 μL (200 μM). (F) NIR-II fluorescence of tumor-to-skin ratio within 72 h after tail vein injection of IR-808, IR-808(RGD), IR-808(RGD)-IP, and IR-808(RGD)-IP2 (n=3). (G) NIR-II imaging of tumor, muscle, and skin of mice *ex vivo* after injecting dyes for 24 h. Exposure time for IR-808 is 5 ms, for IR-808-IP is 15 ms, for IR-808-IP2 is 20 ms, for IR-808(RGD) is 150 ms, for IR-808(RGD)-IP is 40 ms, and for IR-808(RGD)-IP2 is 13 ms. (H) The fluorescence intensity statistics of tumor/skin ratio of mice *ex vivo* from (G).

In addition to achieving regulation of migration time in the lymphatic and vessel system, we further investigated whether this series of dyes could achieve improved tumor-targeting ability. Although it has been widely reported that IR-808 has tumor-seeking capacity due to the *in situ* albumin binding, significant skin uptake severely hampers the ability to distinguish tumors 49. To address this issue, we adopted the as-developed blocking strategy by replacing acetylcysteine with tumor-targeting peptide c(RGDfC) 55, which specifically binds to integrin αvβ3. In this case, RGD served a dual purpose as a blocking group to prevent albumin covalent capture and a targeting group to identify tumors. Meanwhile, the tethered IP moieties can extend the circulation time of the targeting dye, allowing it to specifically bind to the tumor site (**Figure 4A**).

Through base-catalyzed nucleophilic substitution, RGD was successfully connected to dyes (**Figure 4B**). Furthermore, this conjugation did not result insignificant change in the optical properties of the dyes (**Figure S12**). To confirm the αvβ3 integrin specificity of RGD-blocked dyes, we conducted a series of cell imaging experiments. Results verified that prominent fluorescence brightness was observed in αvβ3-positive U87MG cells after incubation with IR-808(RGD), IR-808(RGD)-IP, and IR-808(RGD)-IP2, while negligible fluorescence brightness appeared in the negative group L929 cells (**Figure 4C-D**). We also investigated the tumor accumulation ability of dyes *in vivo*. After intravenous injection of meso-Cl dyes and RGD-blocked dyes into mice bearing U87MG tumors, fluorescence signals were clearly visualized in the tumors over time (**Figure 4E and S13A**), with RGD-blocked dyes showing a much higher signal-to-background ratio (SBR) than dyes without RGD moiety at post-injection time points (**Figure 4F and S13B**). When comparing RGD-blocked dyes modified with different numbers of IPs, the SBR could exceed 4 at 24 h post-injection of IR-808(RGD)-IP and IR-808(RGD)-IP2. Afterward, the tumor, muscle, and skin were surgically excised for NIR fluorescence imaging approximately 24 h post-injection (**Figure 4G, H**). As expected, the nonspecific fluorescence was observed in muscle and skins from meso-Cl dyes-administered cohorts of group, while RGD-blocked dyes showed a satisfactory SBR with extremely low nonspecific fluorescence.

## Conclusion

In summary, we developed a universal strategy for cyanine dyes to regulate their *in vivo* pharmacokinetic behavior affected by different numbers of IP as albumin-binding moieties to alter their pharmacokinetics. Compared to Cl-containing dyes that form complexes *in situ* through inherent covalent albumin binding, the dynamic binding between IP moieties and albumin effectively retained the original advantages of such complexes but with a much lower background signal and tunable pharmacokinetics. These dyes exhibited excellent fluorescence brightness in the NIR-I/II imaging window and could noninvasively and clearly visualize the blood vascular and lymphatic drainage systems. Furthermore, dyes with multiple IPs showed the ability to bind to multiple albumins, allowing for programmable fluorescence signals to "turn on" at different time intervals *in vivo*. This feature is particularly useful for lymph node differentiation and prolonging the half-life time of high-resolution vascular imaging. Our strategy also involves replacing meso-Cl with amino acids or targeted peptides, which can prevent covalent binding between dyes and albumins while conferring tumor-targeting ability. By combining the dynamic albumin-binding moiety and albumin-escaping moiety, this approach greatly reduces nonspecific fluorescence throughout the body and enhances tumor accumulation. Our method can precisely regulate the interaction ability between exogenous molecules and endogenous proteins, achieving controllable *in vivo* pharmacokinetic behavior. It is expected to provide fundamental principles for designing an ideal delivery system of probes and drug molecules, with potential for future clinical applications 55-57.

## Materials and Methods

### Materials

Unless otherwise noted, chemicals for organic synthesis were ordered from TCI or Energy Chemical and used without further purification. Bovine serum albumin (BSA, ≥ 98%) was purchased from Sigma-Aldrich. Dulbecco's Modified Eagle medium (DMEM, Gibco) was purchased from ThermoFisher Scientific Co., LTD.

### Characterization

All ^1^H-NMR spectra were obtained on Bruker AVANCE III 400 MHz or 500 MHz NMR spectrometers (Q. One Instruments Ltd.). Multiplicity was indicated as follows: s (singlet), d (doublet), t (triplet), m (multiple), dd (doublet of doublets). All coupling constants (J) are reported in Hertz (Hz). Mass spectra were in general recorded on QSTAR Elite (ABI). A UV-3100PC spectrophotometer (MAPADA) with background correction was applied to measure the optical absorption spectra. NIR-II Fluorescence spectrophotometry was performed with an Edinburgh instrument FLS980 fluorescence spectrophotometer. Transmission electron microscopy (TEM) images were acquired through DTM-961002 of JEOL Ltd. Atomic Force Microscopy (AFM) images were acquired through Bruker ICON-XR. Sodium dodecyl sulfate-polyacrylamide gel electrophoresis (SDS-PAGE) was performed on the American BIO-RAD electrophoresis system.

### Gel Electrophoresis Analysis

All gel electrophoresis experiments were performed using dedicated SDS-PAGE kits. Initially, separating gels (12.5%) and stacking gels (4%) were prepared. Subsequently, the prepared dyes with BSA solutions were mixed with protein-loading buffer. The resulting samples were then added to their corresponding gel channels for electrophoresis, with the stacking gel run at 80 V and the separating gel at 150 V, both in an ice water bath. Following electrophoresis, the binding between the protein and the dye was analyzed by detecting the NIR-II fluorescent signal.

### Cell Culture and Cell Staining

Human astroblastoma tumor cells U87MG were purchased from the Shanghai Enzyme Research Biotechnology Co., LTD. Cloning of mouse connective tissue L cell line 929 (L929) was supplied by the Laboratory of Opto-Functional Theranostics in Medicine and Chemistry, First Hospital of Jilin University. Cells were cultured in a Dulbecco's Modified Eagle medium (DMEM) supplemented with 10% FBS and 1% Penicillin-Streptomycin (PS) at 37 °C in a humidified atmosphere containing 5% CO_2_. L929 and U87MG cells were seeded onto the confocal dish at a density of 1×10^4^ units per dish and cultured in DMEM supplemented with 10% FBS and 1% PS. After overnight incubation at 37 °C under 5% CO_2_ atmosphere, the incubation media were replaced with fresh ones for different treatments (DMEM, DMEM+IR-808(RGD), DMEM+IR-808(RGD)-IP, DMEM+IR-808(RGD)-IP2, and DMEM; all DMEM contains FBS). The U87MG cell was incubated in DMEM with 5 μM dyes for 1 h and the L929 cell was incubated in DMEM with 10 μM dyes for 1 h. After co-incubation, the cells were washed three times with PBS to remove any residual dyes and imaged using a home-built NIR-II microscopy system with 808 nm excitation and 1000 nm long-pass filter.

### Animal Experiments

All animal experiments were conducted in accordance with institutional guidelines and were approved by the Animal Ethical Committee of The First Hospital of Jilin University (20210642). Balb/C and C57 mice were procured from Liaoning Changsheng Biotechnology Co., Ltd. Balb/C nude mice were procured from Beijing Vital River Laboratory Animal Technology Co., Ltd. All mice were provided with ad libitum access to bedding, nesting materials, food, and water. The ambient temperature was maintained at 20 to 24 °C with a 12 h light/12 h dark cycle. To establish the orthotopic mammary tumor model, ~2 × 10^6^ U87MG cells were inoculated into the flank of the mice.

### NIR-II Imaging

The mice were shaved using Nair hair removal cream and anesthetized with isoflurane. For NIR-II imaging, an 808-nm laser (Artemis Intelligent Imaging) was used as the excitation source with a power density of 65 mW/cm^2^. Fluorescence emission was typically collected using a combination of 1000 and 1100 nm (or 1200, 1300 nm) long-pass filters (Thorlabs, Edmund optics, etc.). The InGaAs camera (Princeton Instruments NIRvana-640 and Raptor) utilized a tunable exposure time to capture images in the NIR-II window.

## Supplementary Material

Experimental details for characterizations, and synthesis details of involved cyanine dyes; experimental protocol of gel electrophoresis analysis, cell experiments, NIR-II bioimaging; absorption and fluorescence spectra of dyes; macromolecular docking results; the tube brightness and electrophoresis analysis of IR-808, IR-808-IP, and IR-808-IP2 with BSA at different incubation temperatures; metabolism of partial dyes; the complete process of vascular imaging and lymphatic imaging.

## Abbreviations

NIR: near-infrared
IP: 4-(4-iodophenyl) butanoic acid
ABM: albumin-binding moiety
ACQ: aggregation-caused quenching effect
PEG: polyethylene glycol
TICT: twisted intramolecular charge transfer
FAs: fatty acids
EB: Evans Blue
Kd: dissociation constants
LN: lymph node
Ac: acetylcysteine
RGD: c(RGDfC)
PBS: phosphate buffered saline

## References

[1] Zhu S, Tian R, Antaris AL, Chen X, Dai H (2019). Near-Infrared-II Molecular Dyes for Cancer Imaging and Surgery. Adv Mater.

[2] Vahrmeijer AL, Hutteman M, van der Vorst JR, van de Velde CJ, Frangioni JV (2013). Image-guided cancer surgery using near-infrared fluorescence. Nat Rev Clin Oncol.

[3] Lei Z, Zhang F (2021). Molecular Engineering of NIR-II Fluorophores for Improved Biomedical Detection. Angew Chem Int Ed Engl.

[4] Park J-E, Kim M, Hwang J-H, Nam J-M (2017). Golden Opportunities: Plasmonic Gold Nanostructures for Biomedical Applications based on the Second Near-Infrared Window. Small Methods.

[5] Tang X, Ren J, Wei X, Wang T, Li H, Sun Y (2023). Exploiting synergistic effect of CO/NO gases for soft tissue transplantation using a hydrogel patch. Nat Commun.

[6] Zhang F, Xu J, Yue Y, Wang Y, Sun J, Song D (2023). Three-dimensional histological electrophoresis enables fast automatic distinguishment of cancer margins and lymph node metastases. Sci Adv.

[7] Hong G, Antaris AL, Dai H (2017). Near-infrared fluorophores for biomedical imaging. Nat Biomed Eng.

[8] Wang Y, Nan J, Ma H, Xu J, Guo F, Wang Y (2023). NIR-II Imaging and Sandwiched Plasmonic Biosensor for Ultrasensitive Intraoperative Definition of Tumor-Invaded Lymph Nodes. Nano Lett.

[9] Wang F, Wan H, Ma Z, Zhong Y, Sun Q, Tian Y (2019). Light-sheet microscopy in the near-infrared II window. Nat Methods.

[10] Chen H, Liu L, Qian K, Liu H, Wang Z, Gao F (2022). Bioinspired large Stokes shift small molecular dyes for biomedical fluorescence imaging. Sci Adv.

[11] Qiu Q, Chang T, Wu Y, Qu C, Chen H, Cheng Z (2022). Liver injury long-term monitoring and fluorescent image-guided tumor surgery using self-assembly amphiphilic donor-acceptor NIR-II dyes. Biosens Bioelectron.

[12] Feng Z, Bai S, Qi J, Sun C, Zhang Y, Yu X (2021). Biologically Excretable Aggregation-Induced Emission Dots for Visualizing Through the Marmosets Intravitally: Horizons in Future Clinical Nanomedicine. Adv Mater.

[13] Fan X, Xia Q, Liu S, Zheng Z, Zhang Y, Wu T (2022). NIR-II and visible fluorescence hybrid imaging-guided surgery via aggregation-induced emission fluorophores cocktails. Mater Today Bio.

[14] Du Y, Liu X, Zhu S (2021). Near-Infrared-II Cyanine/Polymethine Dyes, Current State and Perspective. Front Chem.

[15] Shindy HA (2017). Fundamentals in the chemistry of cyanine dyes: A review. Dyes Pigm.

[16] Usama SM, Burgess K (2021). Hows and Whys of Tumor-Seeking Dyes. Acc Chem Res.

[17] Mei J, Huang Y, Tian H (2018). Progress and Trends in AIE-Based Bioprobes: A Brief Overview. ACS Appl Mater Interfaces.

[18] Tao Z, Hong G, Shinji C, Chen C, Diao S, Antaris AL (2013). Biological imaging using nanoparticles of small organic molecules with fluorescence emission at wavelengths longer than 1000 nm. Angew Chem Int Ed Engl.

[19] Yang Y, Sun C, Wang S, Yan K, Zhao M, Wu B (2022). Counterion-Paired Bright Heptamethine Fluorophores with NIR-II Excitation and Emission Enable Multiplexed Biomedical Imaging. Angew Chem Int Ed Engl.

[20] Cosco ED, Arus BA, Spearman AL, Atallah TL, Lim I, Leland OS (2021). Bright Chromenylium Polymethine Dyes Enable Fast, Four-Color *In vivo* Imaging with Shortwave Infrared Detection. J Am Chem Soc.

[21] Li D, Wang S, Lei Z, Sun C, El-Toni AM, Alhoshan MS (2019). Peroxynitrite Activatable NIR-II Fluorescent Molecular Probe for Drug-Induced Hepatotoxicity Monitoring. Anal Chem.

[22] Antaris AL, Chen H, Cheng K, Sun Y, Hong G, Qu C (2016). A small-molecule dye for NIR-II imaging. Nat Mater.

[23] He Y, Wang S, Yu P, Yan K, Ming J, Yao C (2021). NIR-II cell endocytosis-activated fluorescent probes for *in vivo* high-contrast bioimaging diagnostics. Chem Sci.

[24] Yao C, Chen Y, Zhao M, Wang S, Wu B, Yang Y (2022). A Bright, Renal-Clearable NIR-II Brush Macromolecular Probe with Long Blood Circulation Time for Kidney Disease Bioimaging. Angew Chem Int Ed Engl.

[25] Zeng C, Tan Y, Sun L, Long Y, Zeng F, Wu S (2023). Renal-Clearable Probe with Water Solubility and Photostability for Biomarker-Activatable Detection of Acute Kidney Injuries via NIR-II Fluorescence and Optoacoustic Imaging. ACS Appl Mater Interfaces.

[26] Ganson NJ, Povsic TJ, Sullenger BA, Alexander JH, Zelenkofske SL, Sailstad JM (2016). Pre-existing anti-polyethylene glycol antibody linked to first-exposure allergic reactions to pegnivacogin, a PEGylated RNA aptamer. J Allergy Clin Immunol.

[27] Zhang P, Jain P, Tsao C, Yuan Z, Li W, Li B (2018). Polypeptides with High Zwitterion Density for Safe and Effective Therapeutics. Angew Chem Int Ed Engl.

[28] Zhang P, Sun F, Liu S, Jiang S (2016). Anti-PEG antibodies in the clinic: Current issues and beyond PEGylation. J Control Release.

[29] Tian R, Zeng Q, Zhu S, Lau J, Chandra S, Ertsey R (2019). Albumin-chaperoned cyanine dye yields superbright NIR-II fluorophore with enhanced pharmacokinetics. Sci Adv.

[30] Antaris AL, Chen H, Diao S, Ma Z, Zhang Z, Zhu S (2017). A high quantum yield molecule-protein complex fluorophore for near-infrared II imaging. Nat Commun.

[31] Tian R, Feng X, Wei L, Dai D, Ma Y, Pan H (2022). A genetic engineering strategy for editing near-infrared-II fluorophores. Nat Commun.

[32] Xu J, Han T, Wang Y, Zhang F, Li M, Bai L (2022). Ultrabright Renal-Clearable Cyanine-Protein Nanoprobes for High-Quality NIR-II Angiography and Lymphography. Nano Lett.

[33] Xu Y, Yang C, Wu Y, Jiang W, Cheng Q, Yan L (2023). *In situ* Albumin-Hitchhiking NIR-II Probes for Accurate Detection of Micrometastases. Nano Lett.

[34] Bai L, Hu Z, Han T, Wang Y, Xu J, Jiang G (2022). Super-stable cyanine@albumin fluorophore for enhanced NIR-II bioimaging. Theranostics.

[35] Xing P, Niu Y, Mu R, Wang Z, Xie D, Li H (2020). A pocket-escaping design to prevent the common interference with near-infrared fluorescent probes *in vivo*. Nat Commun.

[36] Liu Z, Chen X (2016). Simple bioconjugate chemistry serves great clinical advances: albumin as a versatile platform for diagnosis and precision therapy. Chem Soc Rev.

[37] Tian R, Zhu S, Zeng Q, Lang L, Ma Y, Kiesewetter DO (2019). An Albumin Sandwich Enhances *in vivo* Circulation and Stability of Metabolically Labile Peptides. Bioconjug Chem.

[38] Sleep D, Cameron J, Evans LR (2013). Albumin as a versatile platform for drug half-life extension. Biochim Biophys Acta.

[39] Liu H, Moynihan KD, Zheng Y, Szeto GL, Li AV, Huang B (2014). Structure-based programming of lymph-node targeting in molecular vaccines. Nature.

[40] Curry S, Mandelkow H, Brick P, Franks N (1998). Crystal structure of human serum albumin complexed with fatty acid reveals an asymmetric distribution of binding sites. Nat Struct Biol.

[41] Wu C, Yue X, Lang L, Kiesewetter DO, Li F, Zhu Z (2014). Longitudinal PET imaging of muscular inflammation using 18F-DPA-714 and 18F-Alfatide II and differentiation with tumors. Theranostics.

[42] Zhu G, Lynn GM, Jacobson O, Chen K, Liu Y, Zhang H (2017). Albumin/vaccine nanocomplexes that assemble *in vivo* for combination cancer immunotherapy. Nat Commun.

[43] Fu H, Huang J, Zhao T, Wang H, Chen Y, Xu W (2023). Fibroblast Activation Protein-Targeted Radioligand Therapy with 177Lu-EB-FAPI for Metastatic Radioiodine-Refractory Thyroid Cancer: First-in-Human, Dose-Escalation Study. Clin Cancer Res.

[44] Dumelin CE, Truessel S, Buller F, Trachsel E, Bootz F, Zhang Y (2008). A portable albumin binder from a DNA-encoded chemical library. Angew Chem Int Ed Engl.

[45] Truessel S, Dumelin C, Frey K, Villa A, Buller F, Neri D (2009). New Strategy for the Extension of the Serum Half-Life of Antibody Fragments. Bioconjug Chem.

[46] Kelly JM, Amor-Coarasa A, Nikolopoulou A, Wustemann T, Barelli P, Kim D (2017). Dual-Target Binding Ligands with Modulated Pharmacokinetics for Endoradiotherapy of Prostate Cancer. J Nucl Med.

[47] Zorzi A, Linciano S, Angelini A (2019). Non-covalent albumin-binding ligands for extending the circulating half-life of small biotherapeutics. Medchemcomm.

[48] Shi C, Wu JB, Pan D (2016). Review on near-infrared heptamethine cyanine dyes as theranostic agents for tumor imaging, targeting, and photodynamic therapy. J Biomed Opt.

[49] Yang X, Shi C, Tong R, Qian W, Zhau HE, Wang R (2010). Near IR heptamethine cyanine dye-mediated cancer imaging. Clin Cancer Res.

[50] Usama SM, Park GK, Nomura S, Baek Y, Choi HS, Burgess K (2020). Role of Albumin in Accumulation and Persistence of Tumor-Seeking Cyanine Dyes. Bioconjug Chem.

[51] Thavornpradit S, Usama SM, Lin CM, Burgess K (2019). Protein labelling and albumin binding characteristics of the near-IR Cy7 fluorophore, QuatCy. Org Biomol Chem.

[52] Canovas C, Bellaye P-S, Moreau M, Romieu A, Denat F, Goncalves V (2018). Site- specific near- infrared fluorescent labelling of proteins on cysteine residues with meso- chlorosubstituted heptamethine cyanine dyes. Org Biomol Chem.

[53] Rao DA, Forrest ML, Alani AWG, Kwon GS, Robinson JR (2010). Biodegradable PLGA Based Nanoparticles for Sustained Regional Lymphatic Drug Delivery. J Pharm Sci.

[54] Gomi M, Sakurai Y, Okada T, Miura N, Tanaka H, Akita H (2021). Development of Sentinel LN Imaging with a Combination of HAase Based on a Comprehensive Analysis of the Intra-lymphatic Kinetics of LPs. Mol Ther.

[55] Tsai W-K, Wang C-I, Liao C-H, Yao C-N, Kuo T-J, Liu M-H (2019). Molecular design of near-infrared fluorescent Pdots for tumor targeting: aggregation-induced emission versus anti-aggregation-caused quenching. Chem Sci.

[56] Long Y-T, Meade TJ (2020). Advances in optical and electrochemical techniques for biomedical imaging. Chem Sci.

[57] Zhang W, Kohane DS (2021). Keeping Nanomedicine on Target. Nano Lett.

