# Albumin-seeking dyes with adjustable assemblies *in situ* enable programmable imaging windows and targeting tumor imaging: Erratum

**DOI:** 10.7150/thno.126837

**Published:** 2026-01-01

**Authors:** Yijing Du, Jiajun Xu, Tianyang Han, Zijian Jiang, Yuewei Zhang, Jia Li, Xiaoyuan Chen, Shoujun Zhu

**Affiliations:** 1 Joint Laboratory of Opto-Functional Theranostics in Medicine and Chemistry, First Hospital of Jilin University, Jilin University, Changchun 130021, P.R. China.; 2 State Key Laboratory of Supramolecular Structure and Materials, Center for Supramolecular Chemical Biology, College of Chemistry, Jilin University, Changchun 130012, P.R. China.; 3 School of Chemistry and Pharmaceutical Engineering, Jilin Institute of Chemical Technology, Jilin 132022, P.R. China.; 4 Departments of Diagnostic Radiology, Surgery, Chemical and Biomolecular Engineering, Biomedical Engineering, Yong Loo Lin School of Medicine and College of Design and Engineering, National University of Singapore, 117597, Singapore, Singapore.; 5 Clinical Imaging Research Centre, Centre for Translational Medicine, Yong Loo Lin School of Medicine, National University of Singapore, Singapore 117599, Singapore.; 6 Nanomedicine Translational Research Program, NUS Center for Nanomedicine, Yong Loo Lin School of Medicine, National University of Singapore, Singapore 117609, Singapore.

The authors deeply regret that in the original version of Figure 3A, one of the NIR-II images at the 10-minute time point in the IR-808-IP group was mistakenly replaced with the image from the same time point in the IR-808 group. This error occurred during data processing: after acquisition, all images were processed using ImageJ for uniform brightness intensity and compiled in PowerPoint for layout. Since images from two groups (IR-808 and IR-808-IP) were both labeled “10” and appeared similar, a mistake was made during the copy-and-paste process. The same mistakes also occurred in Figure 2G and Figure S9A. We have re-examined the original experimental data, corrected the misplaced image, and have uploaded all original data for verification.

Furthermore, we have added the TEM images that were missing from Figure 1D and F, which may have been lost during the publication process.

This correction does not affect any of the results or conclusions of the study. The authors apologize for any inconvenience that these errors may have caused.

## Figures and Tables

**Figure A:**
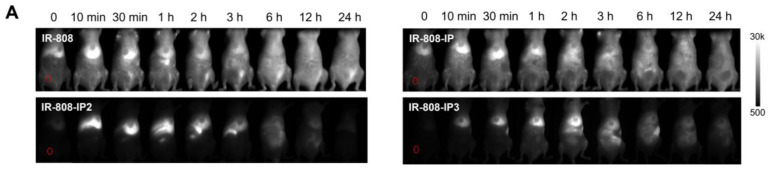
Corrected Figure 3A.

**Figure B:**
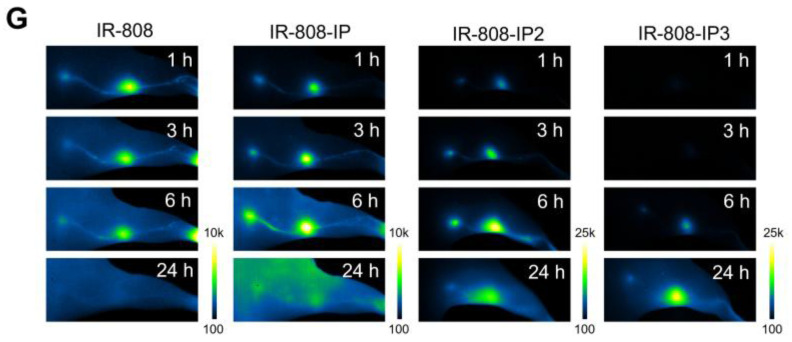
Corrected Figure 2G.

**Figure C:**
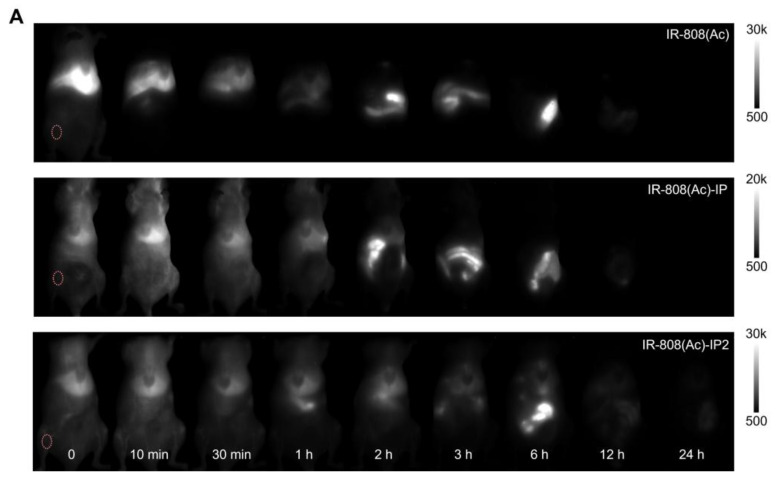
Corrected Figure S9.

**Figure D:**
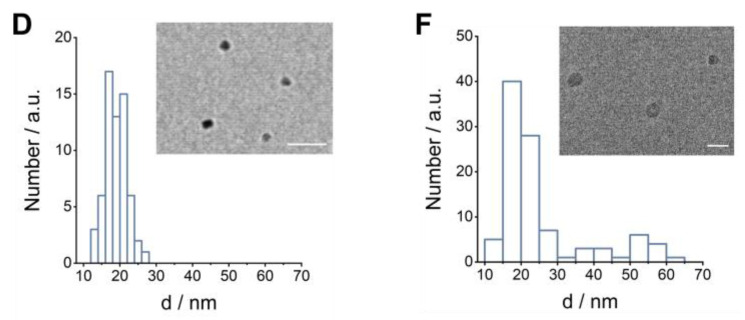
Corrected Figure 1D and F.

