# Supplementary material for: Albumin-seeking dyes with adjustable assemblies in situ enable programmable imaging windows and targeting tumor imaging
Source: Theranostics. 2024 Apr 15;14(7):2675–86. doi: 10.7150/thno.92991 (PMC11103493; doi:10.7150/thno.92991)
Supplement: Supplementary file 1 — Experimental details for characterizations, and synthesis details of involved cyanine dyes; experimental protocol of gel electrophoresis analysis, cell experiments, NIR-II bioimaging; absorption and fluorescence spectra of dyes; macromolecular docking results; the tube brightness and electrophoresis analysis of IR-808, IR-808-IP, and IR-808-IP2 with BSA at different incubation temperatures; metabolism of partial dyes; the complete process of vascular imaging and lymphatic imaging. [file thnov14p2675s1.pdf]

Supplementary material

Supplementary Figures and Tables

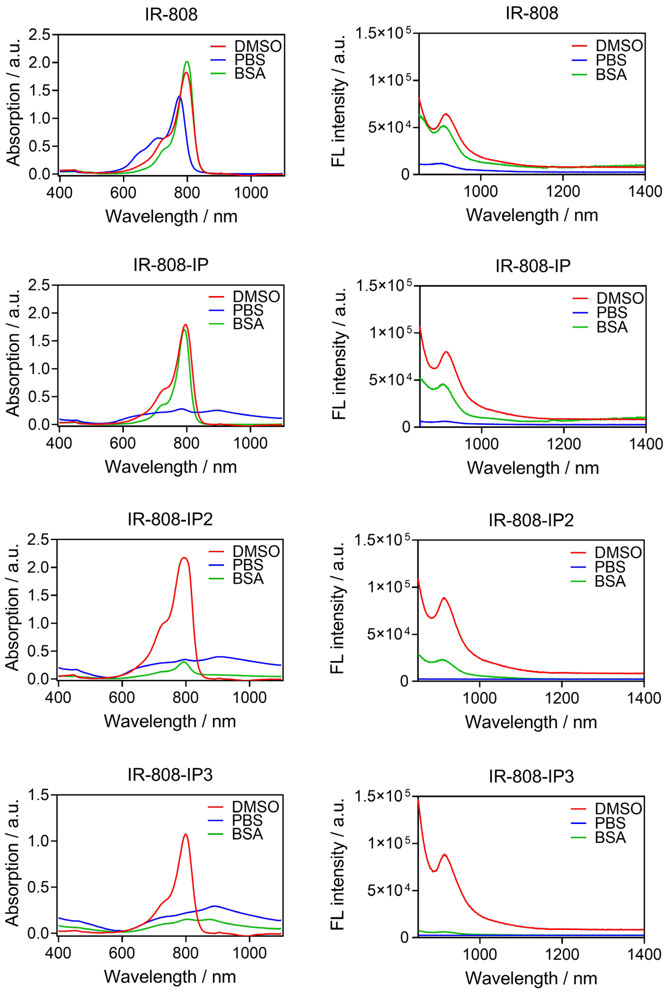

Figure S1. Absorption spectra and fluorescence (FL) spectra of IR-808, IR-808-IP, IR-808-IP2, and IR-808-IP3 in DMSO, PBS, and BSA respectively. Concentration of dyes: 10 μM.

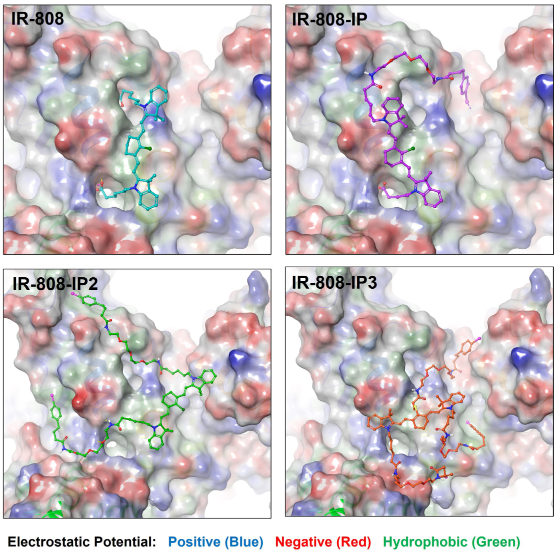

Figure S2. Macromolecular docking results indicated that different binding abilities and conformations were shown between different dyes and albumin.

Table S1. The docking score and binding energy of IR-808, IR-808-IP, IR-808-IP2, and IR-808-IP3.

Ligand	Docking Score （kcal/mol)	Binding Energy （kcal/mol)
IR-808	-7.05	-43.07
IR-808-IP	-5.67	-33.48
IR-808-IP2	-5.30	-26.87
IR-808-IP3	-4.94	-14.95

Note: Macromolecular docking using Glide program, and Binding energy calculation using MM-GBSA.

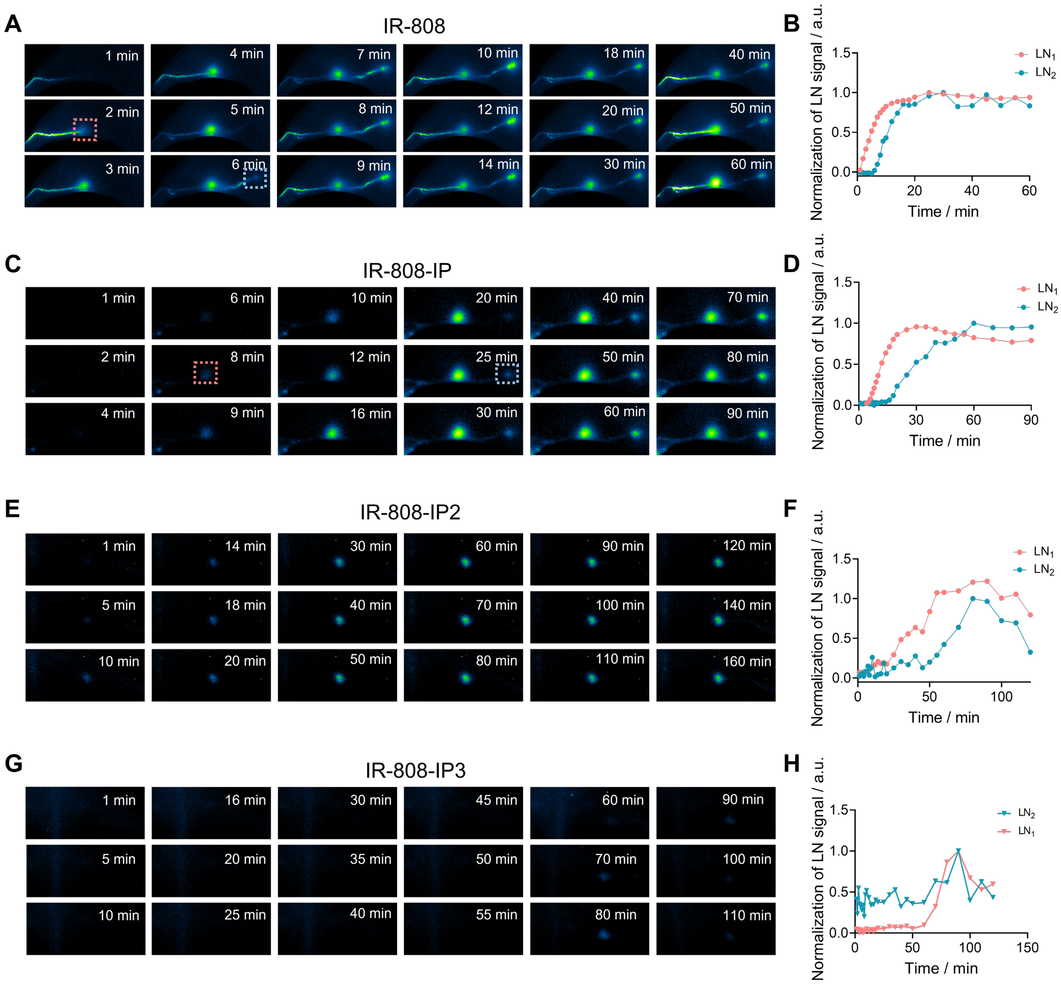

Figure S3. (A-H) Selected time points from NIR-II imaging of lymph nodes after intradermal injection of IR-808, IR-808-IP, IR-808-IP2, and IR-808-IP3 in the footpad of shaved Balb/c mice. The normalization of LN fluorescence intensity by the highest intensity is shown on the right. The fluorescence intensity of IR-808-IP3 was too low to have statistical significance. Excitation: 808 nm with 65 mW/cm^2 power density; Filter: 1000 /1100 nm long-pass filters; All doses of footpad injection were 25 μL (200 μM). Exposure time: IR-808 is 50 ms and the others are 200 ms.

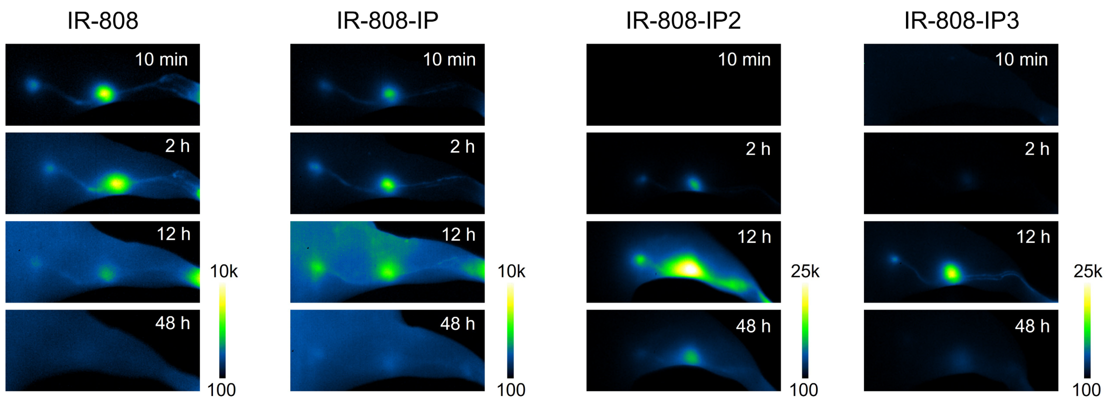

Figure S4. NIR-II fluorescence imaging of mice lymph nodes of remaining time points after intradermal injection of dyes. Laser excitation: 808 nm with 65 mW/cm^2 power density; Over 1100 nm collection; All doses of footpad injection were 25 μL (200 μM). Exposure time for IR-808 is 20 ms, for IR-808-IP and IR-808-IP2 are 60 ms, for IR-808-IP3 is 100 ms.

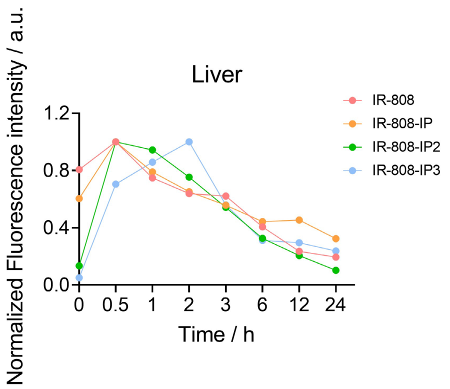

Figure S5. The normalization of fluorescence intensity statistics of mice liver within 24 h after the tail vein injection of IR-808, IR-808-IP, IR-808-IP2, and IR-808-IP3. All doses of the tail vein injection were 200 μL (200 μM).

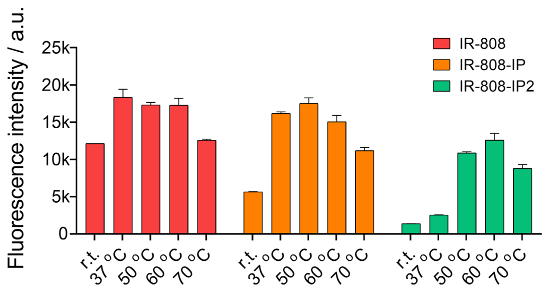

Figure S6. The fluorescence intensity statistics after incubating IR-808, IR-808-IP, and IR-808-IP2 with equal concentrations of BSA in PBS for 2 h at different temperatures. Concentration: 10 μM; Laser excitation: 808 nm with 65 mW/cm^2 power density; Over

1000 nm collection; Exposure time: 3 ms.

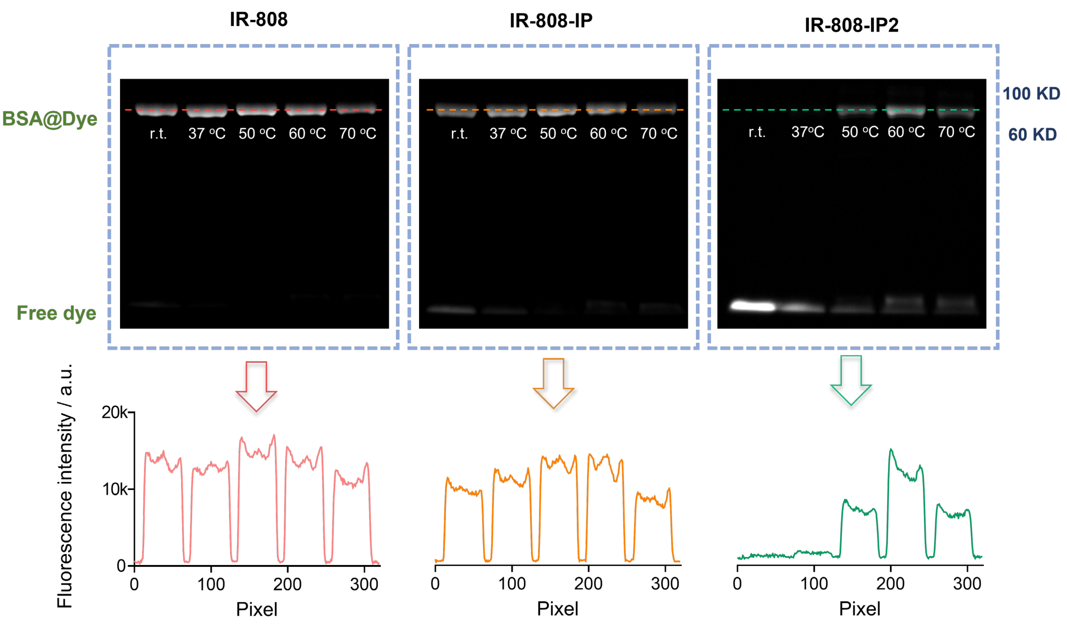

Figure S7. Electrophoresis analysis of the dye@BSA from Figure S6 and the fluorescent cross-sectional intensity profile of the ROI region was plotted in the figure below. The results showed that there was no covalent binding observed for IR-808-IP2 under both room temperature (r.t.) and 37 °C conditions.

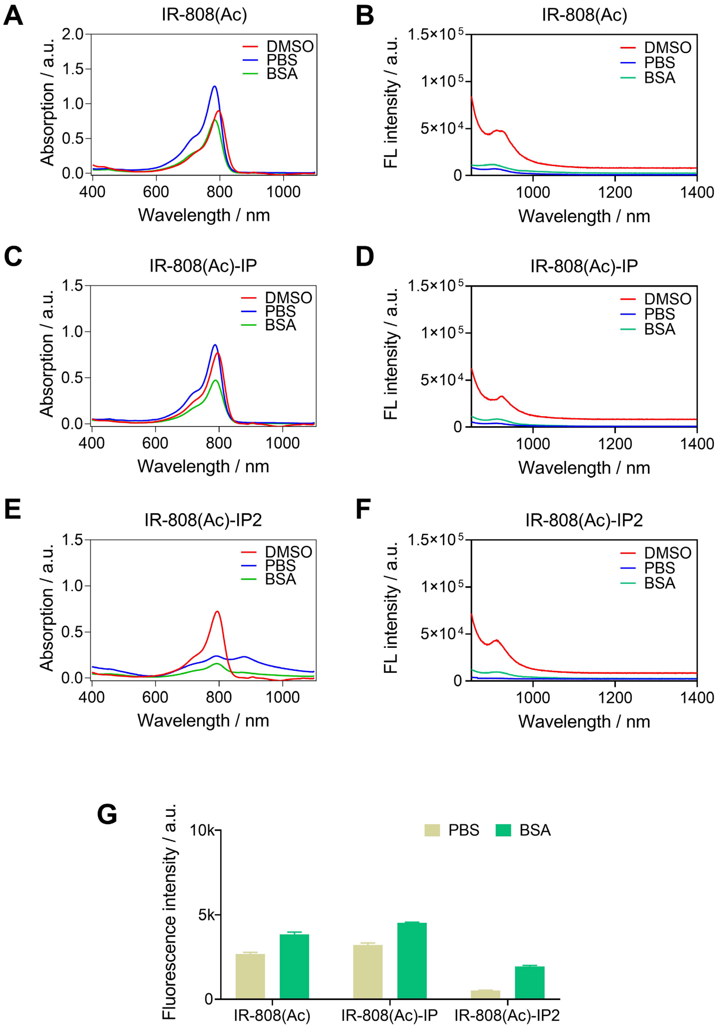

Figure S8. (A-F) Absorption spectra and fluorescence spectra of IR-808(Ac), IR-808(Ac)-IP, and IR-808(Ac)-IP2 in DMSO, PBS, and BSA respectively. Concentration: 10 μM. (G) The Eppendorf tube brightness of dyes in PBS and BSA after equal mole incubation at 37 °C for 2 h. Laser excitation: 808 nm with 65 mW/cm^2; Concentration: 10 μM. Over 1000 nm collection; Exposure time: 3 ms.

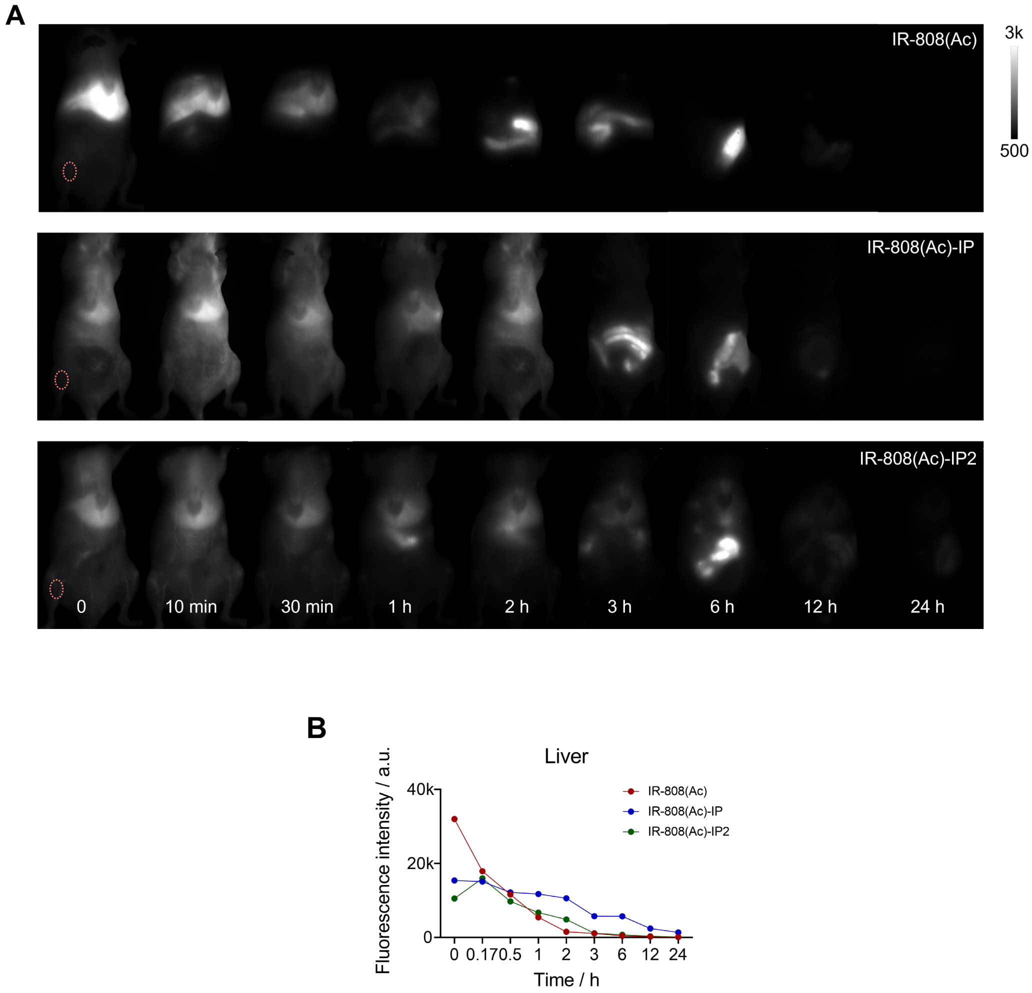

Figure S9. (A) Whole-body NIR-II imaging of mice 0–24 h after tail vein injection of IR-808(Ac), IR-808(Ac)-IP, and IR-808(Ac)-IP2. The red dotted circles are regions of interest (ROIs) of skin. (B) The fluorescence intensity statistics of mice liver within 24 h after the tail vein injection. All doses of the tail vein injection were 200 μL (200 μM). 808 nm excitation with 65 mW/cm^2 power density; Filter: 1000 /1100 nm; Exposure time for IR-808(Ac) and IR-808(Ac)-IP are 30 ms, IR-808(Ac)-IP2 is 100 ms.

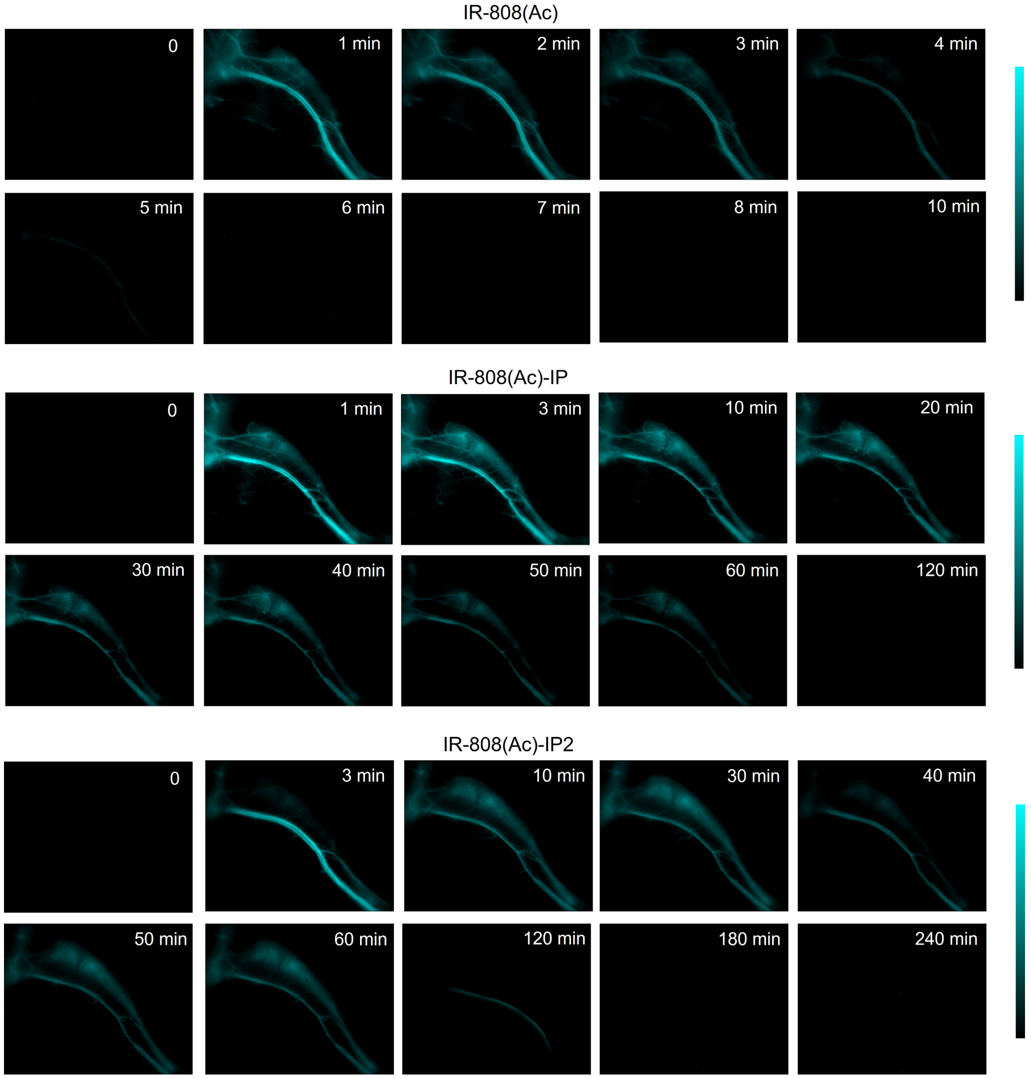

Figure S10. NIR-II fluorescence imaging of mice hind limb blood vessels at selected time points after the tail vein injection of IR-808(Ac), IR-808(Ac)-IP, and IR-808(Ac)-IP2. Results indicated that IP-connected dyes can substantially extend the imaging window. 808 nm excitation with 100 mW/cm^2 power density; Over 1300 nm collection; Injection dose: 200 μL (600 μM). Exposure time for IR-808(Ac) is 15 ms, for IR-808(Ac)-IP is 30 ms and for IR-808(Ac)-IP2 is 100 ms.

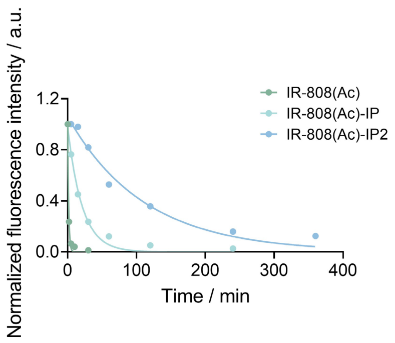

Figure. S11 Normalized fluorescence intensity and exponential fitting curves of collected blood at different time points after tail vein injection of IR-808(Ac), IR-808(Ac)-IP, and IR-808(Ac)-IP2. Injection dose: 200 μL (600 μM). The blood circulation half-time of IR-808(Ac) is 1.3 min, IR-808(Ac)-IP is 18.1 min, and IR-808(Ac)-IP2 is 104.9 min.

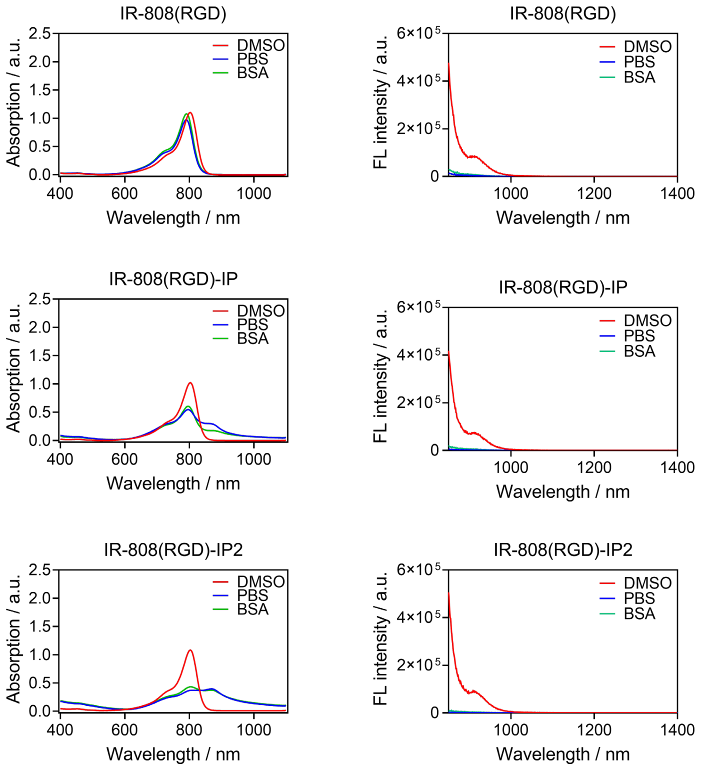

Figure S12. Absorption spectra and fluorescence spectra of IR-808(RGD), IR-808(RGD)-IP, and IR-808(RGD)-IP2 in DMSO, PBS, and BSA respectively. Concentration: 10 μM.

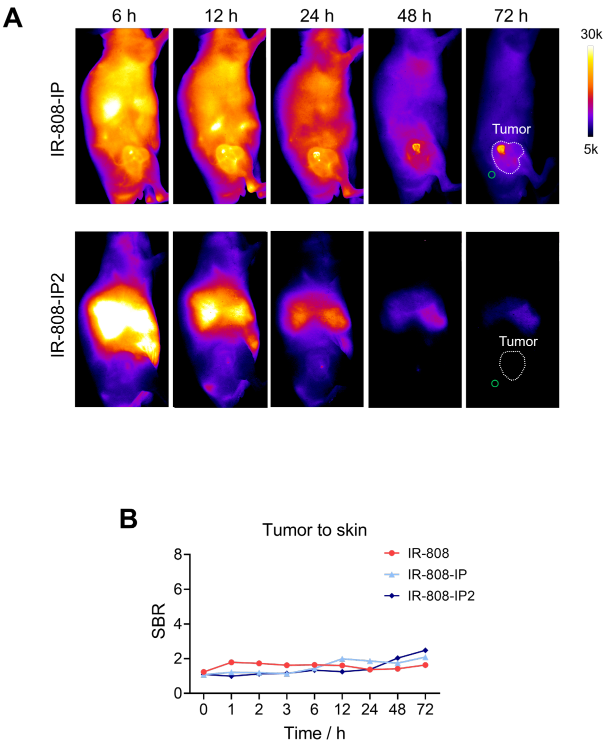

Figure S13. (A) NIR-II imaging of U87MG tumor-bearing mice injected with IR-808-IP and IR-808-IP2 over time. The green circles are ROIs of skin surrounding the tumor site, and the white dotted circles are ROIs of tumor. (B) NIR-II fluorescence of tumor-to-skin ratio within 72 h after tail vein injection of IR-808, IR-808-IP, and IR-808-IP2.

Synthetic Procedures of Cyanine Dyes

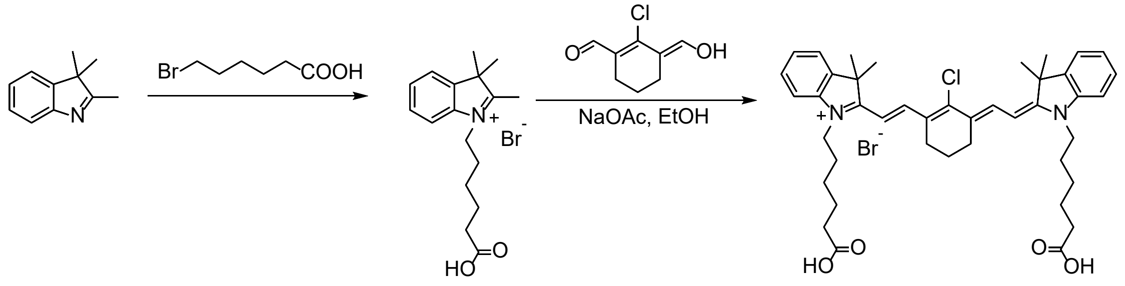

1 2 IR-808

Synthesis of 1-(5-carboxypentyl)-2,3,3-trimethyl-3H-indol-1-ium (2).[1] A mixture of 2,3,3-trimethylindolenine (1.95 g, 10 mmol), and 6-bromohexanoic acid (1.6 mL, 10 mmol) was heated at 110 °C for 12 h. After cooling to room temperature, the precipitate was washed with acetone three times. The crude product was collected as a pink solid (3.2 g, 74%).

^{1}H-NMR (400 MHz, DMSO-d_6, ppm): δ 8.00 – 7.94 (m, 1H), 7.87 – 7.80 (m, 1H), 7.66 – 7.58 (m, 2H), 4.49 – 4.40 (m, 2H), 2.84 (s, 3H), 2.22 (d, J = 6.8 Hz, 2H), 1.83 (t, J = 7.6 Hz, 2H), 1.61 – 1.48 (m, 8H), 1.43 (t, J = 7.8 Hz, 2H).

Synthesis of IR-808. (E)-2-chloro-3-(hydroxymethylene) cyclohex-1-ene-1-carbaldehyde (43 mg, 0.25 mmol), compound **2** (177.2 mg, 0.5 mmol) and sodium acetate (NaOAc, 41 mg, 0.5 mmol) were dissolved in 2 mL ethanol (EtOH) and stirred at 70 °C for 4 h. The solvent was removed under reduced pressure and purified by column chromatography on silica gel using DCM/MeOH. The yield of green solid was 78% (150 mg).

^{1}H-NMR (400 MHz, $CDCl_3$, ppm): δ 8.34 (d, J = 13.9 Hz, 2H), 7.58 – 7.34 (m, 4H), 7.27 – 7.21 (m, 2H), 7.18 (d, J = 7.9 Hz, 2H), 6.21 (d, J = 14.0 Hz, 2H), 4.19 – 4.10 (m, 4H), 2.80 – 2.67 (m, 4H), 2.51 (t, J = 7.2 Hz, 4H), 2.01 (s, 2H), 1.87 (m, 4H),

1.78 (t, J = 7.3 Hz, 4H), 1.71 (s, 12H), 1.63 – 1.52 (m, 4H).

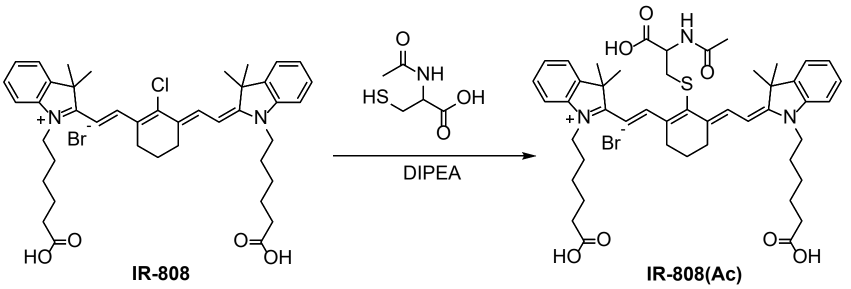

Synthesis of IR-808(Ac).[2] IR-808 (10 mg, 0.013 mmol) and acetylcysteine (5 mg, 0.03 mmol) were dissolved in 1 mL dimethyl sulfoxide (DMSO). Then added 5 μL *N*,*N*-Diisopropylethylamine (DIPEA) and stirred at room temperature for 1 h. The crude product was purified by column chromatography on silica gel using DCM/MeOH. The yield of green solid was 95% (11 mg).

^{1}H-NMR (400 MHz, CD_3OD, ppm): δ 8.79 (d, J = 14.0 Hz, 2H), 7.48 (d, J = 7.4 Hz, 2H), 7.39 (t, J = 7.7 Hz, 2H), 7.34 – 7.20 (m, 4H), 6.24 (d, J = 14.1 Hz, 2H), 4.38 (dd, J = 7.5, 4.0 Hz, 1H), 4.13 (t, J = 7.2 Hz, 4H), 3.32 – 3.29 (m, 2H), 2.71 – 2.56 (m, 4H), 2.19 (t, J = 7.2 Hz, 4H), 1.89 – 1.95 (m, 5H), 1.84 (t, J = 7.5 Hz, 4H), 1.74 (s, 12H), 1.68 (t, J = 7.6 Hz, 4H), 1.49 (q, J = 7.7 Hz, 4H).

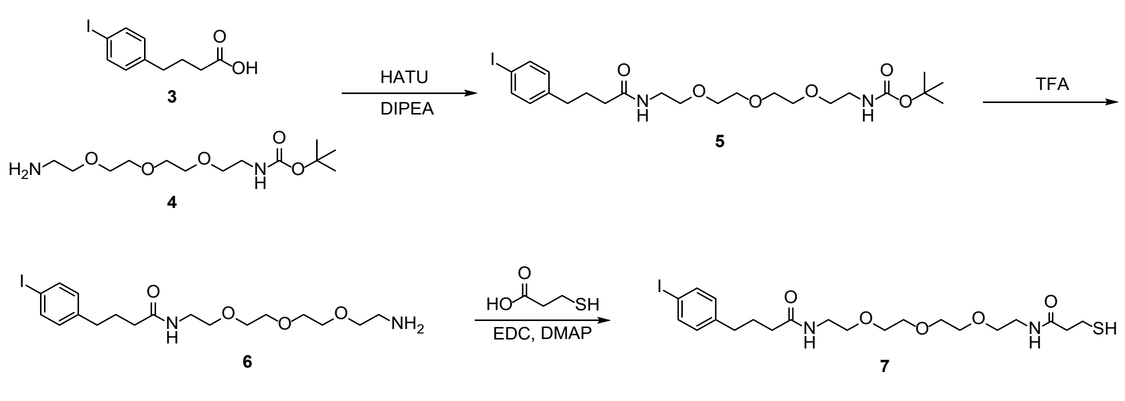

Synthesis of tert-butyl (16-(4-iodophenyl)-13-oxo-3,6,9-trioxa-12-azahexadecyl)carbamate (5). 2-(7-Azabenzotriazol-1-yl)-N,N,N',N'-

tetramethyluronium hexafluorophosphate (HATU, 129 mg, 0.34 mmol) and compound **3** (100 mg, 0.34 mmol) were dissolved in DCM (5 mL) and stirred for 30 min under N_2 atmosphere. Then dropped slowly with the mixture of compound **4** (82 mg, 0.28 mmol) and DIPEA (54 mg, 0.42 mmol) in DCM (5 mL), stirred overnight at room temperature. The mixture was purified by column chromatography on silica gel using EA/Hexane. The yield of pale-yellow solid was 40% (67 mg).

Synthesis of N-(2-(2-(2-(2-aminoethoxy)ethoxy)ethoxy)ethyl)-4-(4-iodophenyl)butanamide (6). Compound 5 (50 mg, 0.1 mmol) was dissolved in DCM (1 mL) and TFA (0.5 mL) was slowly dropped at 0 ºC. After 5 h, the mixture was washed three times with saturated sodium bicarbonate solution and saturated salt solution once, dehydrated using sodium sulfate, and filtered. The DCM solvent was removed by rotary evaporation and the crude product was purified by column chromatography. The yield of pale-yellow solid was 50% (25 mg).

^{1}H-NMR (400 MHz, $CDCl_3$, ppm): δ 7.75 – 7.54 (m, 2H), 6.98 (d, J = 8.0 Hz, 2H), 3.66 (m, 8H), 3.57 (dt, J = 9.8, 5.0 Hz, 4H), 3.47 (q, J = 5.2 Hz, 2H), 2.87 (t, J = 5.1 Hz, 2H), 2.62 (t, J = 7.6 Hz, 2H), 2.21 (t, J = 7.5 Hz, 2H), 1.97 (p, J = 7.5 Hz, 2H).

Synthesis of 4-(4-iodophenyl)-N-(15-mercapto-13-oxo-3,6,9-trioxa-12-azapentadecyl) butanamide (7). 3-mercaptopropionic acid (4.6 mg, 0.043 mmol) and compound **6** (20 mg, 0.043 mmol) were dissolved in 0.1 mL DCM. The flask was cooled in an ice bath, followed by the addition of 1-(3-Dimethylaminopropyl)-3-ethylcarbodiimide hydrochloride (EDC, 10 mg, 0.056 mmol) and 4-dimethylaminopyridine (DMAP, 1 mg, 0.01 mmol). The mixture was stirred for a

further 12 h. Then the mixture was washed three times with a saturated aqueous solution of citric acid, dehydrated using sodium sulfate, and filtered. The DCM solvent was removed by rotary evaporation and the crude product was purified by column chromatography using EA/Hexane.

^{1}H-NMR (400 MHz, $CDCl_3$, ppm): δ 7.63 (d, J = 8.2 Hz, 2H), 6.97 (d, J = 8.0 Hz, 2H), 3.66 (d, J = 6.2 Hz, 8H), 3.59 (t, J = 5.2 Hz, 4H), 3.49 (p, J = 4.8 Hz, 4H), 2.84 (dt, J = 8.2, 6.7 Hz, 2H), 2.63 (t, J = 7.5 Hz, 2H), 2.53 (t, J = 6.8 Hz, 2H), 2.20 (t, J = 7.5 Hz, 2H), 1.97 (p, J = 7.5 Hz, 2H), 1.67 (t, J = 8.3 Hz, 1H), 1.29 (s, 1H).

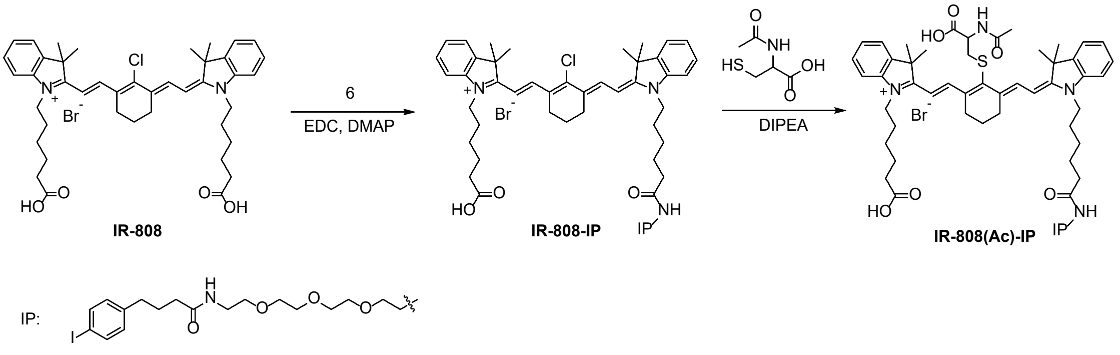

Synthesis of IR-808-IP. IR-808 (10 mg, 0.012 mmol) and compound **6** (7 mg, 0.012 mmol) were dissolved in 1 mL DCM. The flask was cooled in an ice bath, followed by the addition of EDC (7 mg, 0.024 mmol) and DMAP (0.5 mg, 0.004 mmol). The mixture was stirred for a further 4 h. Then the mixture was washed three times with a saturated aqueous solution of citric acid, dehydrated using sodium sulfate, and filtered. The DCM solvent was removed by rotary evaporation and the crude product was purified by column chromatography. The crude product was purified by column chromatography on silica gel using DCM/MeOH.

^{1}H-NMR (400 MHz, CD_3OD, ppm): δ 8.43 (dd, J = 14.1, 4.3 Hz, 2H), 8.19 – 8.08

(m, 1H), 7.57 (d, *J* = 8.2 Hz, 2H), 7.53 (dt, *J* = 7.5, 1.7 Hz, 2H), 7.48 – 7.40 (m, 2H), 7.31 (ddt, *J* = 18.0, 7.4, 3.6 Hz, 4H), 6.99 (d, *J* = 8.5 Hz, 1H), 6.97 (d, *J* = 8.2 Hz, 2H), 6.29 (dd, *J* = 14.1, 5.7 Hz, 2H), 4.19 (td, *J* = 7.4, 4.0 Hz, 4H), 3.69 – 3.58 (m, 6H), 3.59 – 3.54 (m, 2H), 3.49 (dt, *J* = 7.8, 5.4 Hz, 4H), 3.37 – 3.29 (m, 4H), 2.73 (t, *J* = 5.9 Hz, 4H), 2.56 (t, *J* = 7.6 Hz, 2H), 2.30 (t, *J* = 7.3 Hz, 2H), 2.20 (dt, *J* = 14.6, 7.4 Hz, 4H), 1.91 – 1.81 (m, 5H), 1.73 (s, *J* = 1.8 Hz, 12H), 1.71 – 1.62 (m, 4H), 1.60 – 1.41 (m, 4H), 1.28 (s, 2H).

Synthesis of IR-808(Ac)-IP. IR-808-IP (5 mg, 0.004 mmol) and acetylcysteine (2 mg, 0.012 mmol) were dissolved in 1 mL dimethyl sulfoxide (DMSO). Then added 2 μL *N*,*N*-Diisopropylethylamine (DIPEA) and stirred at room temperature for 1 h. The crude product was purified by column chromatography on silica gel using DCM/MeOH. The yield of green solid was 93% (5.2 mg).

^{1}H-NMR (400 MHz, CD_3OD, ppm): δ 8.84 (d, *J* = 14.0 Hz, 2H), 7.61 (d, *J* = 8.3 Hz, 2H), 7.54 (d, *J* = 7.2 Hz, 2H), 7.48 – 7.38 (m, 2H), 7.36 – 7.23 (m, 4H), 7.07 – 6.99 (m, 2H), 6.29 (dd, *J* = 14.1, 10.7 Hz, 2H), 4.43 (d, *J* = 3.7 Hz, 1H), 4.19 (q, *J* = 8.4, 7.6 Hz, 4H), 3.66 – 3.62 (m, 6H), 3.61 – 3.58 (m, 2H), 3.54 (d, *J* = 5.7 Hz, 2H), 3.52 – 3.49 (m, 2H), 3.38 – 3.34 (m, 4H), 3.33 (d, *J* = 1.7 Hz, 2H), 2.99 – 2.90 (m, 4H), 2.59 (t, *J* = 7.6 Hz, 2H), 2.30 (t, *J* = 7.1 Hz, 2H), 2.27 – 2.16 (m, 4H), 1.98 (s, 3H), 1.95 – 1.85 (m, 6H), 1.79 (s, 12H), 1.76 – 1.65 (m, 4H), 1.50 (d, *J* = 15.1 Hz, 4H), 1.32 (s, 2H).

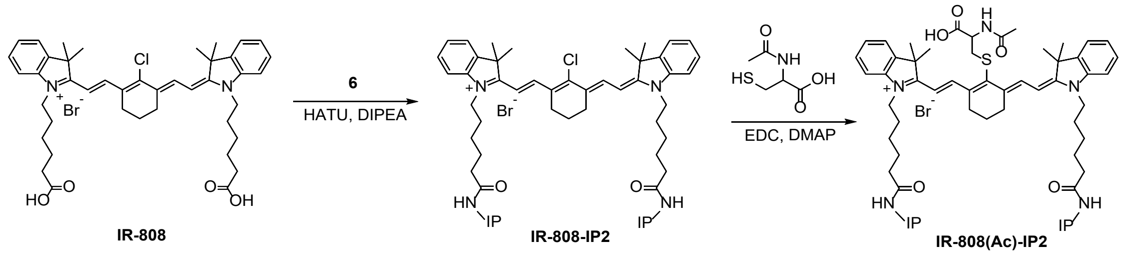

Synthesis of IR-808-IP2. HATU (2.47 mg, 0.0065 mmol) and IR-808 (5 mg, 0.0065 mmol) were dissolved in DCM and stirred for 30 min under N_2 atmosphere. Then dropped slowly with the mixture of compound **6** (6 mg, 0.013 mmol) and DIPEA (1 mg, 0.008 mmol) in DCM, stirred overnight at room temperature. The DCM solvent was removed by rotary evaporation and the crude product was purified by column chromatography on silica gel using DCM/MeOH.

^{1}H-NMR (400 MHz, CD_3OD, ppm): δ 8.46 (d, J = 13.8 Hz, 2H), 7.61 (d, J = 7.9 Hz, 4H), 7.56 (d, J = 7.4 Hz, 2H), 7.47 (t, J = 7.6 Hz, 2H), 7.39 – 7.25 (m, 4H), 7.00 (d, J = 7.9 Hz, 4H), 6.32 (d, J = 13.9 Hz, 2H), 4.21 (s, 4H), 3.67 – 3.61 (m, 12H), 3.60 (s, 6H), 3.54 (d, J = 5.7 Hz, 4H), 3.53 – 3.49 (m, 4H), 3.39 – 3.35 (m, 6H), 2.76 (s, 4H), 2.59 (t, J = 7.6 Hz, 4H), 2.23 (dt, J = 14.7, 7.2 Hz, 8H), 1.93 – 1.86 (m, 8H), 1.76 (s, 12H), 1.71 (d, J = 7.3 Hz, 4H), 1.50 (d, J = 8.6 Hz, 4H), 1.32 (s, 2H).

Synthesis of IR-808(Ac)-IP2. IR-808-IP2 (5 mg, 0.0065 mmol) and acetylcysteine (1 mg, 0.013 mmol) were dissolved in 1 mL DMSO. Then added 5 μL *N*,*N*-Diisopropylethylamine (DIPEA) and stirred at room temperature for 1 h. The crude product was purified by column chromatography on silica gel using DCM/MeOH.

^{1}H-NMR (500 MHz, CD_3OD, ppm): δ 8.87 – 8.69 (m, 2H), 7.60 (dd, J = 8.5, 2.0 Hz, 4H), 7.53 (d, J = 7.5 Hz, 2H), 7.47 – 7.38 (m, 2H), 7.34 – 7.25 (m, 4H), 7.07 – 6.96 (m, 4H), 6.27 (d, J = 14.1 Hz, 2H), 4.43 (dd, J = 7.0, 4.4 Hz, 1H), 4.16 (t, J = 7.4 Hz,

4H), 3.62 (qd, J = 4.3, 3.9, 1.5 Hz, 12H), 3.60 – 3.57 (m, 4H), 3.52 (dt, J = 8.7, 5.5 Hz, 8H), 3.35 (dd, J = 5.5, 2.3 Hz, 6H), 3.33 (p, J = 1.6 Hz, 2H), 2.67 (q, J = 6.7 Hz, 4H), 2.59 (t, J = 7.6 Hz, 4H), 2.22 (dt, J = 16.5, 7.4 Hz, 8H), 2.03 (d, J = 6.9 Hz, 2H), 1.97 (s, 3H), 1.89 (m, J = 7.5 Hz, 8H), 1.78 (s, 12H), 1.72 (p, J = 7.5 Hz, 4H), 1.54 – 1.45 (m, 4H), 1.36 – 1.27 (m, 2H).

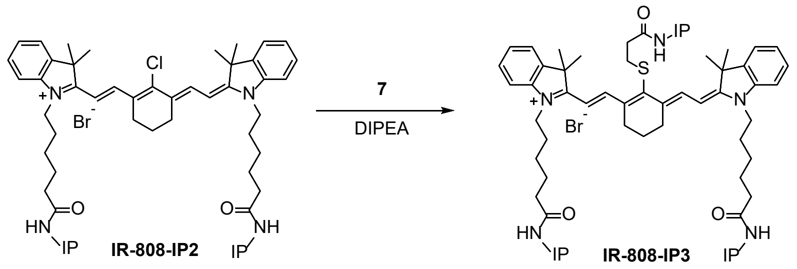

Synthesis of IR-808-IP3. IR-808-IP2 (5 mg, 0.0065 mmol) and compound **7** (10 mg, 0.0195 mmol) were dissolved in 1 mL DMSO. Then added 1 μL *N,N*-Diisopropylethylamine (DIPEA) and stirred at room temperature for 1 h. The crude product was purified by column chromatography on silica gel using DCM/MeOH.

NMR Spectra and MS data of the Synthesized Compounds

^{1}H NMR of **compound 2**

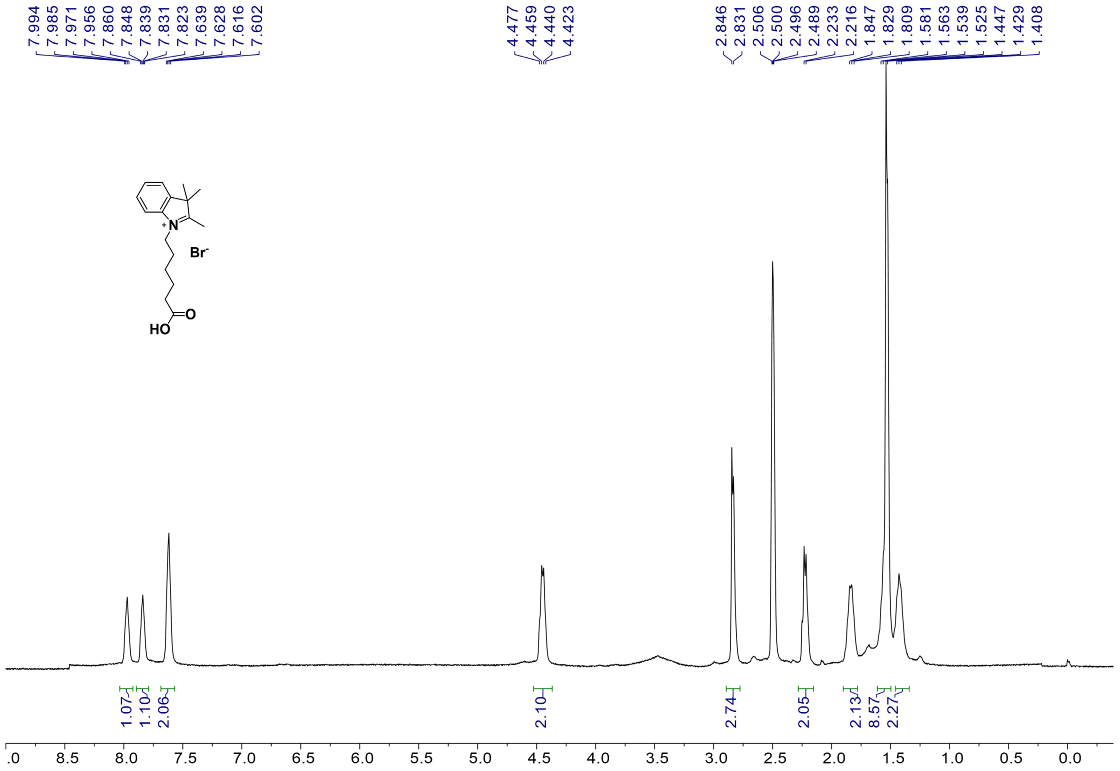

^{1}H NMR of **IR-808**

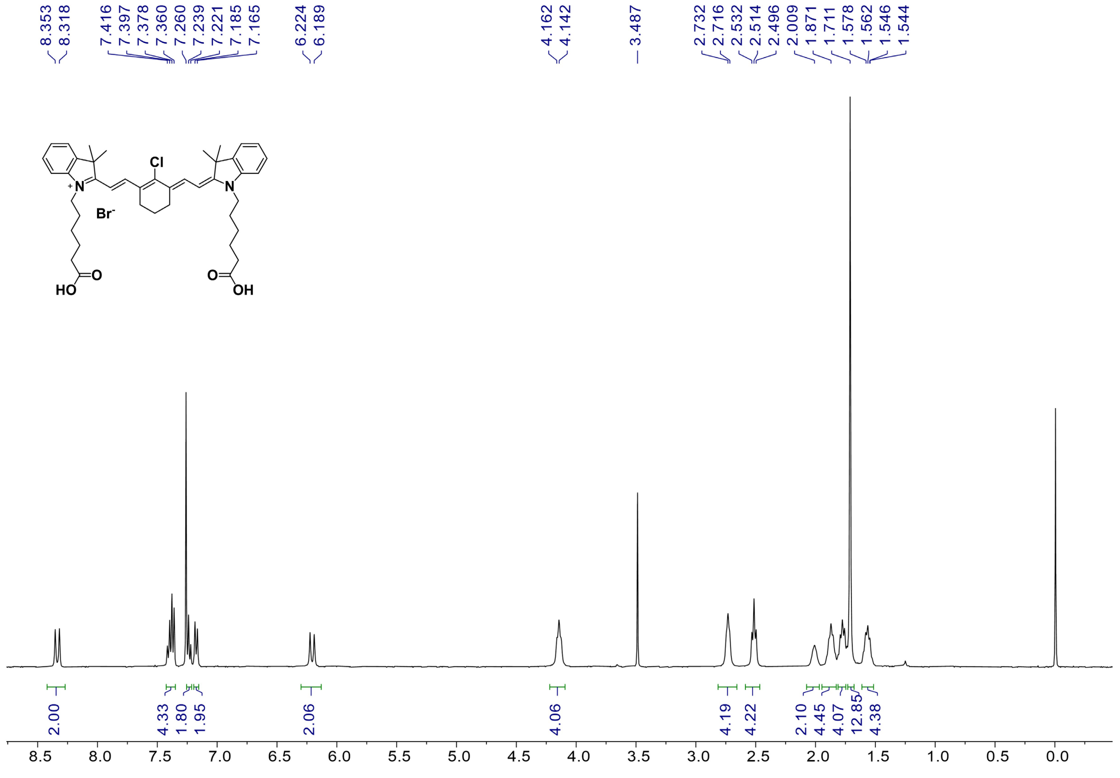

^{1}H NMR of **IR-808(Ac)**

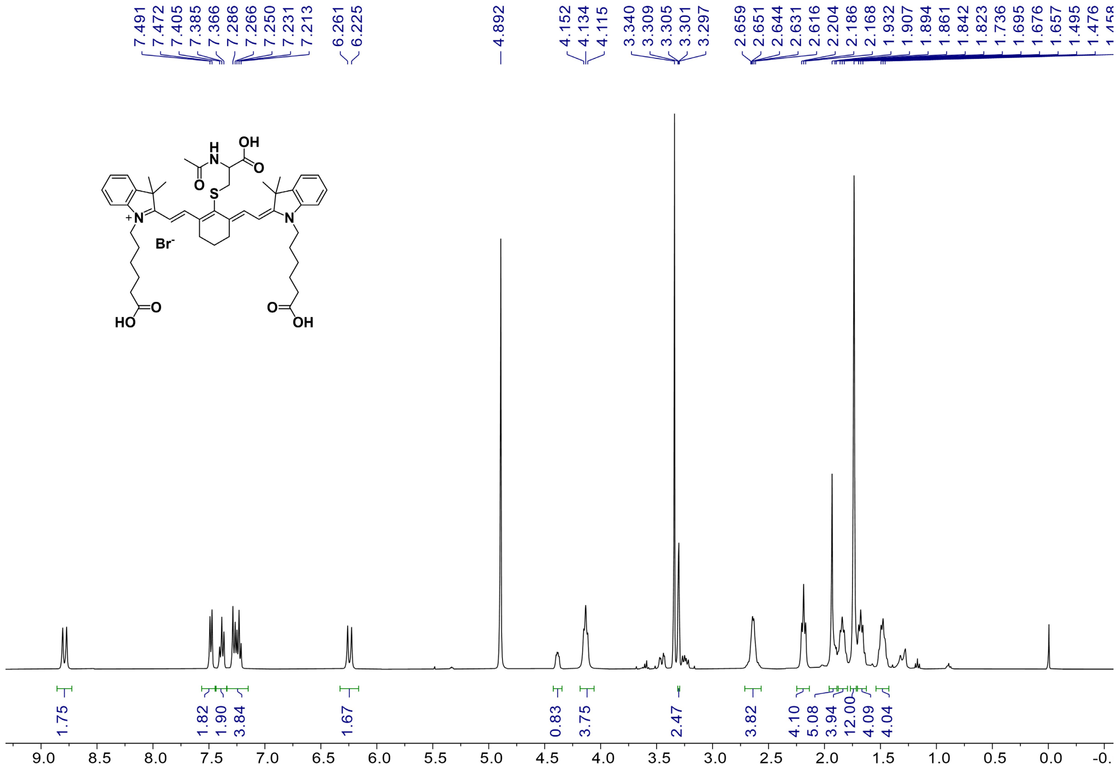

^{1}H NMR of **compound 6**

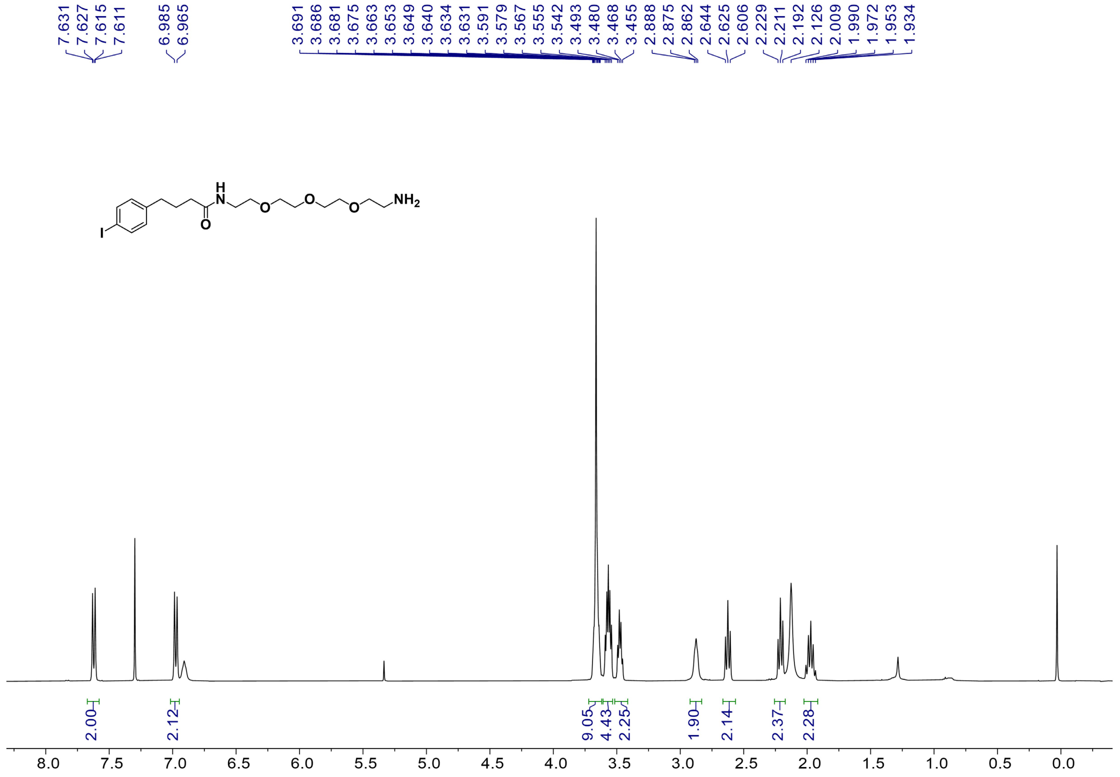

^{1}H NMR of **compound 7**

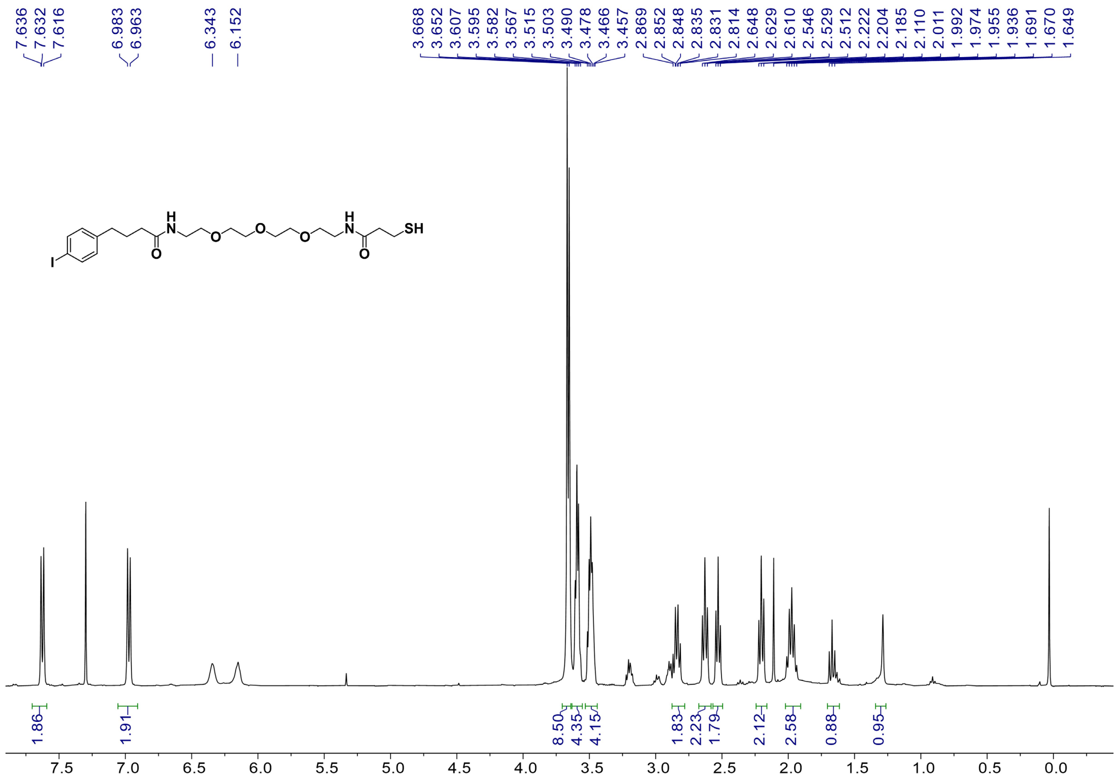

^{1}H NMR of **IR-808-IP**

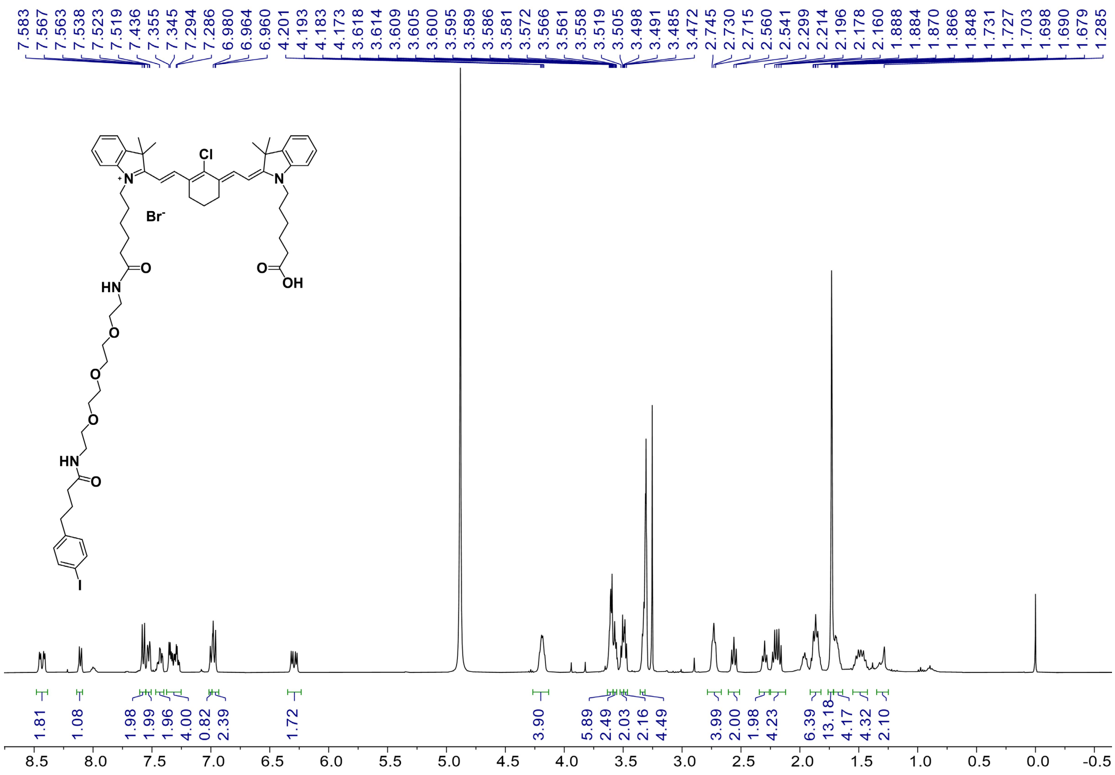

^{1}H NMR of **IR-808(Ac)-IP**

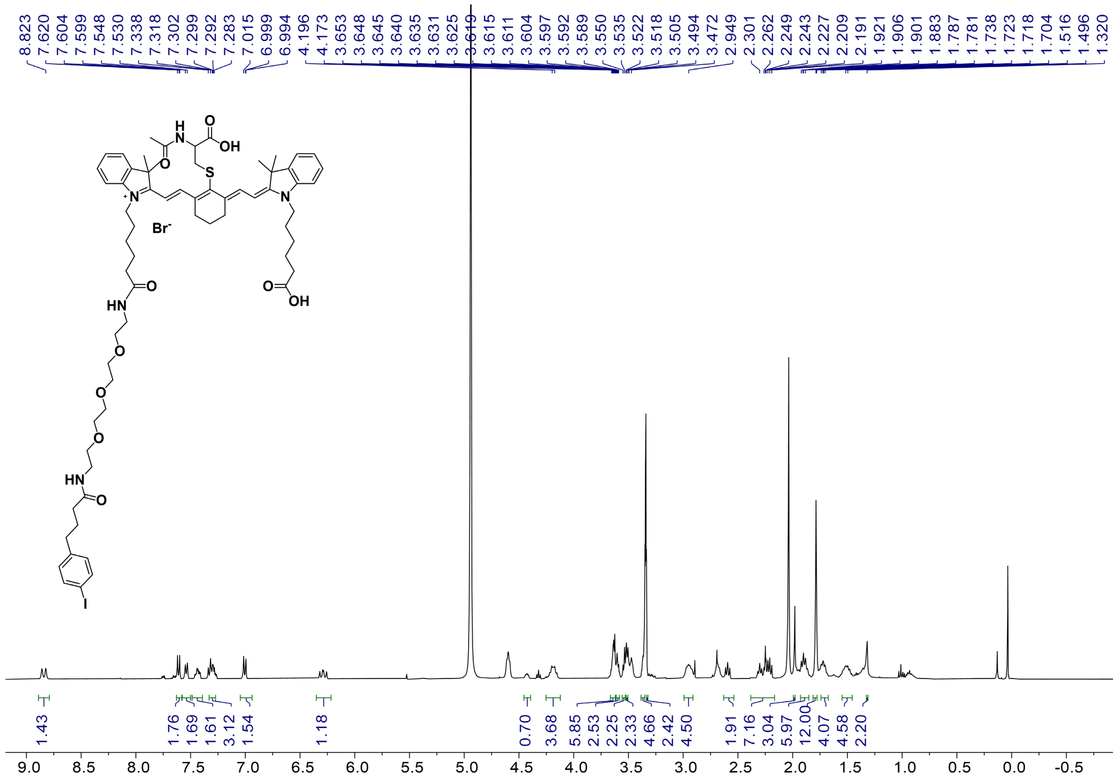

^{1}H NMR of **IR-808-IP2**

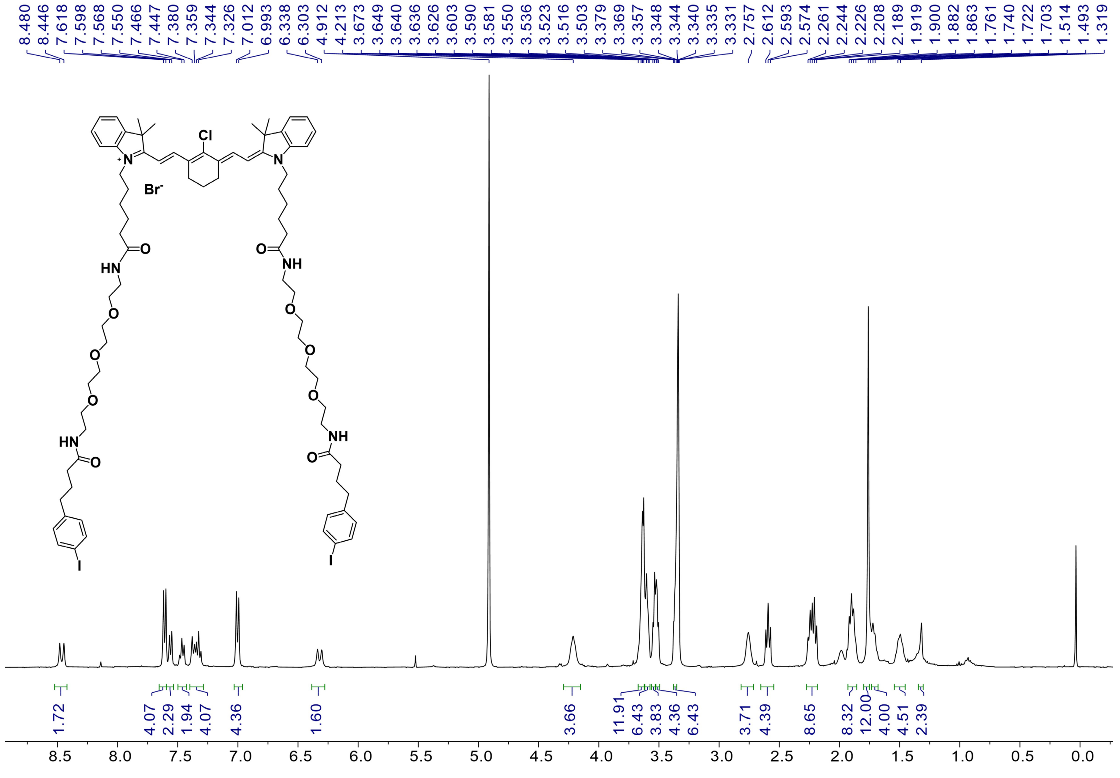

^{1}H NMR of **IR-808(Ac)-IP2**

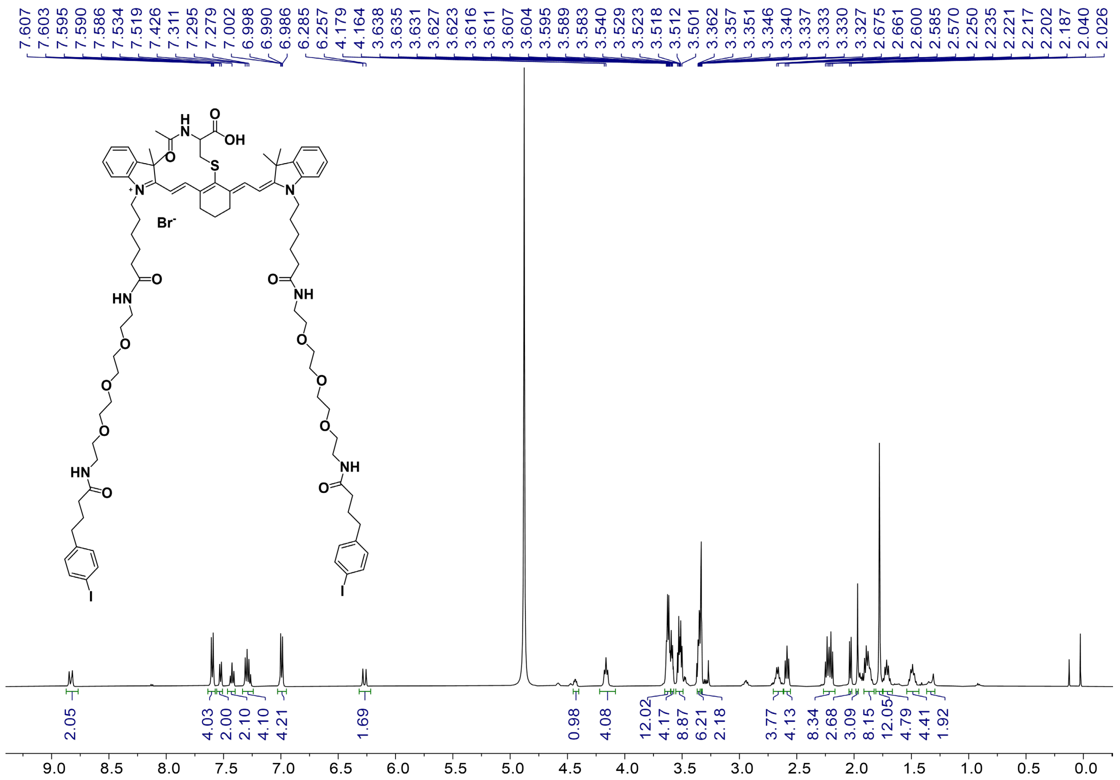

MS spectra of the synthesized compounds

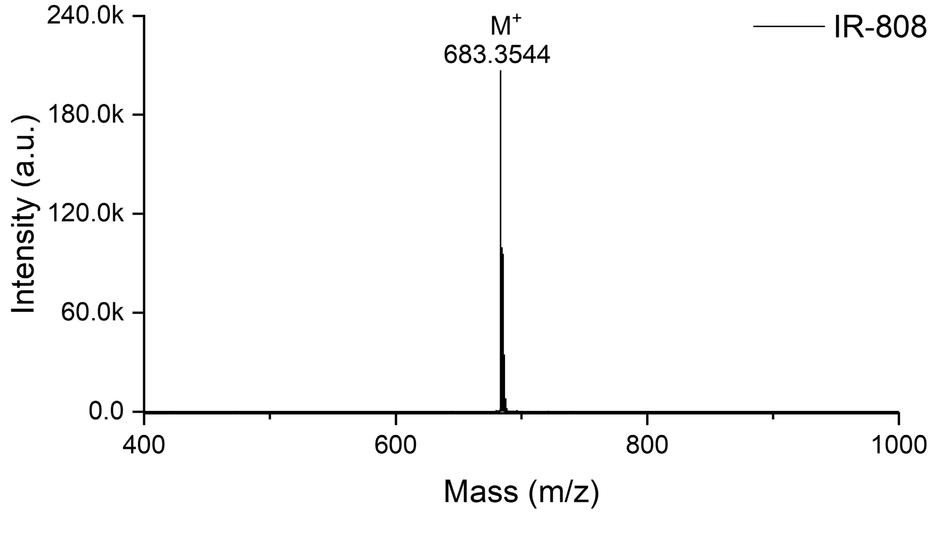

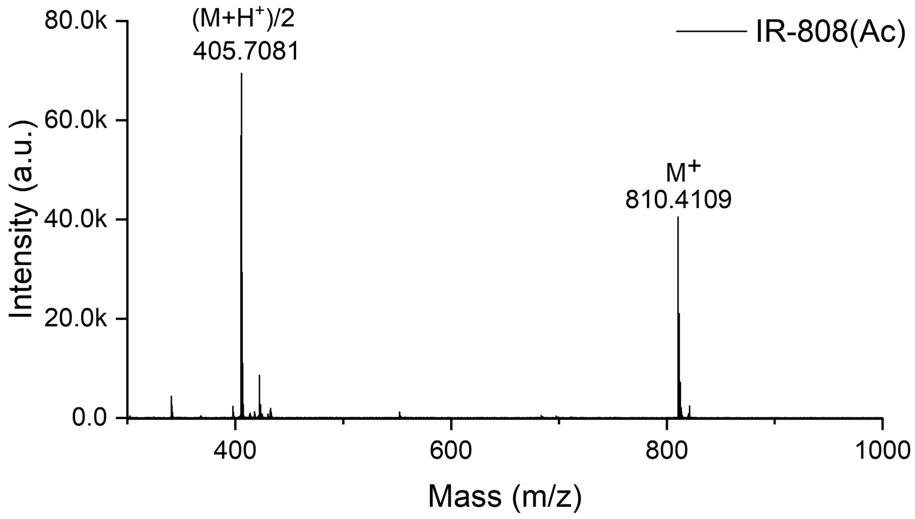
IR-808(Ac)
(M+H+)/2
405.7081
M+
810.4109
Intensity (a.u.)
Mass (m/z)

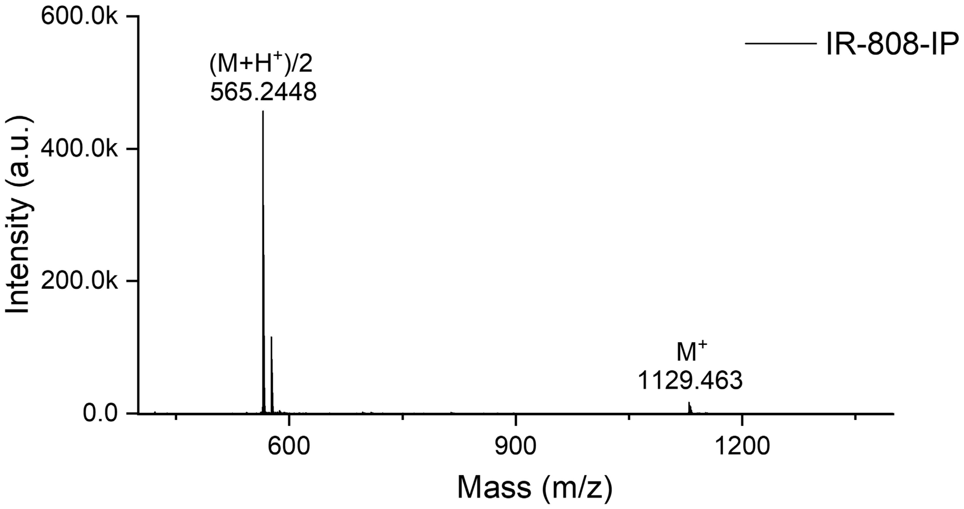
IR-808-IP
(M+H+)/2
565.2448
M+
1129.463
Intensity (a.u.)
Mass (m/z)

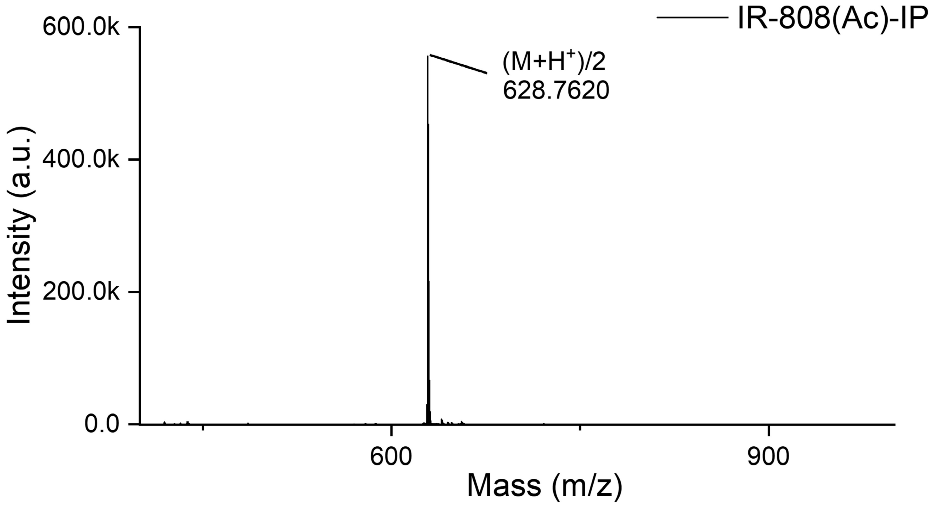
IR-808(Ac)-IP
(M+H+)/2
628.7620
Intensity (a.u.)
Mass (m/z)

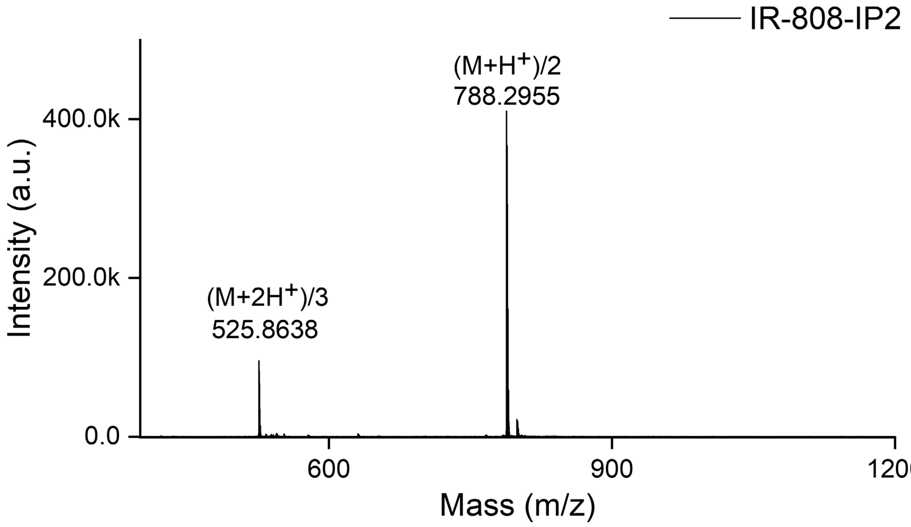
IR-808-IP2
(M+H+)/2
788.2955
(M+2H+)/3
525.8638
Intensity (a.u.)
400.0k
200.0k
0.0
600
900
1200
Mass (m/z)

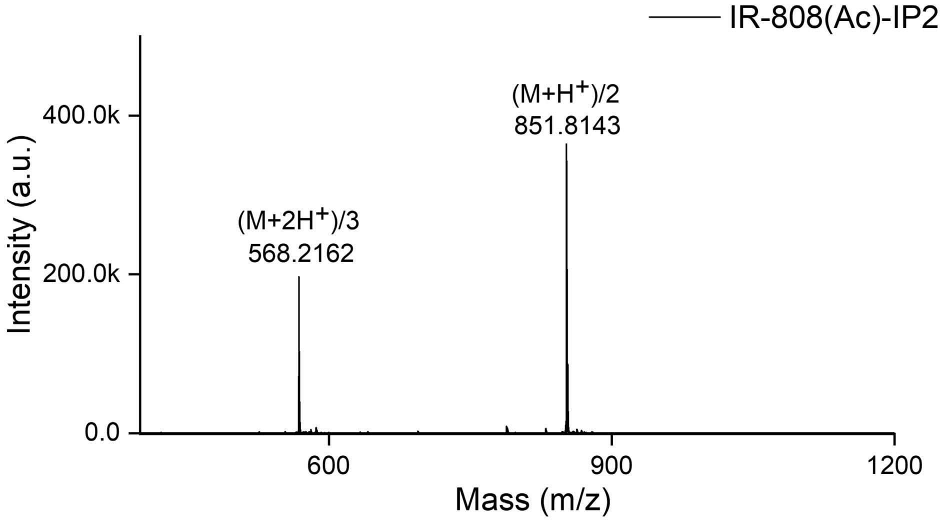
IR-808(Ac)-IP2
(M+H+)/2
851.8143
(M+2H+)/3
568.2162
Intensity (a.u.)
400.0k
200.0k
0.0
600
900
1200
Mass (m/z)

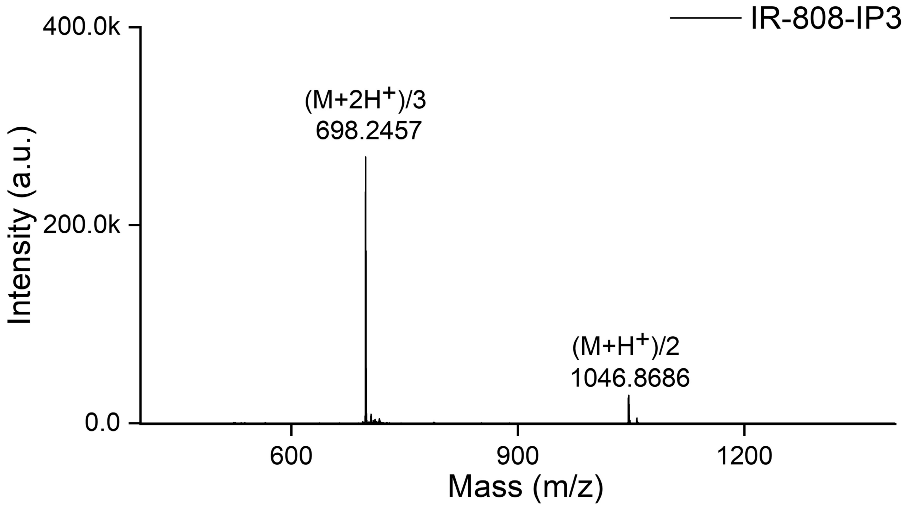
IR-808-IP3
(M+2H+)/3
698.2457
(M+H+)/2
1046.8686
Intensity (a.u.)
400.0k
200.0k
0.0
600
900
1200
Mass (m/z)

References

1 S. M. Usama, G. K. Park, S. Nomura, Y. Baek, H. S. Choi and K. Burgess, Role of Albumin in Accumulation and Persistence of Tumor-Seeking Cyanine Dyes. Bioconjug Chem. 2020; **31**: 248-259.

2 S. Thavornpradit, S. M. Usama, C. M. Lin and K. Burgess, Protein labelling and albumin binding characteristics of the near-IR Cy7 fluorophore, QuatCy. Org Biomol Chem. 2019; **17**: 7150-7154.
